# High potency of lipid conjugated TLR7 agonist requires nanoparticulate or liposomal formulation

**DOI:** 10.1016/j.ejps.2018.07.048

**Published:** 2018-10-15

**Authors:** Adam J.R. Gadd, Valeria Castelletto, Elena Kabova, Kenneth Shankland, Yvonne Perrie, Ian Hamley, Alexander J.A. Cobb, F. Greco, Alexander D. Edwards

**Affiliations:** a School of Chemistry, Food and Pharmacy, University of Reading, Whiteknights, Reading RG6 6AD, United Kingdom; b Strathclyde Institute of Pharmacy and Biomedical Sciences, University of Strathclyde, Glasgow G1 1XQ, United Kingdom; c Department of Chemistry, 7 Trinity Street, King's College London, London SE1 1DB, United Kingdom

**Keywords:** TLR7 agonist, Lipid conjugate, Liposomes, Conjugation, Immunostimulatory

## Abstract

Conjugation of small molecule agonists of Toll-like receptor 7 (TLR7) to proteins, lipids, or polymers is known to modulate potency, and the physical form or formulation of these conjugates is likely to have a major effect on their immunostimulatory activity. Here, we studied the effect of formulation on potency of a 1,2‑di‑(9Z‑octadecenoyl)‑*sn*‑*glycero*‑3‑phosphoethanolamine (DOPE) conjugated TLR7 agonist (DOPE-TLR7a) alongside assessing physical form using Dynamic Light Scattering (DLS), Nanosight Particle Tracking (NTA) analysis and Small Angle X-ray Scattering (SAXS). A very high potency of DOPE-TLR7a conjugate (EC_50_ around 9 nM) was observed either when prepared by direct dilution from DMSO or when formulated into 400–700 nm large multilamella liposomes containing dimethyldioctadecylammonium bromide salt (DDA) and DOPE. When prepared by dissolution in DMSO followed by dilution in aqueous culture medium, 93 ± 5 nm nanoparticles were formed. Without dilution from solution in DMSO, no nanoparticles were observed and no immunostimulatory activity could be detected without this formulation step. SAXS analysis of the conjugate after DMSO dissolution/water dilution revealed a lamellar order with a layer spacing of 68.7 Å, which correlates with arrangement in groups of 3 bilayers. The addition of another immunostimulatory glycolipid, trehalose‑6,6‑dibehenate (TDB), to DOPE:DDA liposomes gave no further increase in immunostimulatory activity beyond that provided by incorporating DOPE-TLR7a. Given the importance of nanoparticle or liposomal formulation for activity, we conclude that the major mechanism for increased potency when TLR7 agonists are conjugated to macromolecules is through alteration of physical form.

## Graphical abstract

**Figure:**
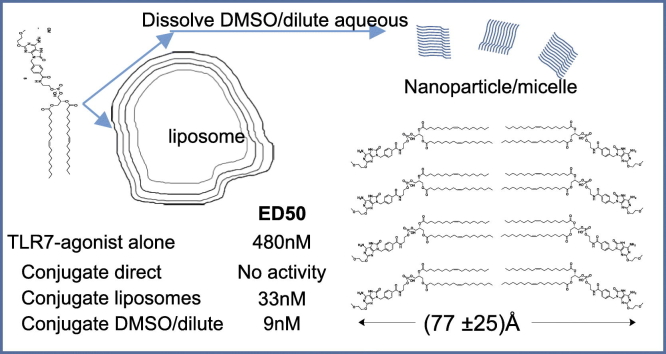

## 1 Introduction

Recently the molecular pathways of immune activation both by pathogen associated molecular patterns (PAMP) recognition *via* pattern recognition receptors (PRR) such as TLR (Medzhitov and Janeway, 1998; Medzhitov et al., 1997; Takeda and Akira, 2004) and also by particulate adjuvants for example by nucleotide-binding domain, leucine-rich-containing family, pyrin domain-containing-3 (NLRP3)/inflammasome mediated pathways (Eisenbarth et al., 2008) have been elucidated. These advances include insight into the immunomodulatory properties of alum, which was thought to rely on a deposition effect to enhance the persistence of presented antigens to antigen presenting cells (APC) (Verdier et al., 2005). However this paradigm has been challenged by studying the rapid release of ^14^C labelled antigens administered with alum (Gupta et al., 1996) and the excision of the injection site shortly after administration does not significantly impact on the immune response to the antigen (Lindblad, 2004). The increased immunogenicity of alum adsorbed antigen may therefore lie in the efficiency of APC to phagocytise particulate antigens compared to the macropinocytosis of antigen alone despite the lack of PRR agonist (Morefield et al., 2005). The physical form of the antigen is therefore clearly of equal importance to associated immunostimulatory activity through PRR. Recent attempts at rational vaccine design therefore manipulate both the innate molecular recognition pathways and the physical form of the antigen. For example, when a small molecule TLR7/8 agonist was conjugated to a temperature responsive polymer support to form nanoparticles, the most significant increase in immunogenicity was seen from the formation of particles as opposed to modulation of TLR7/8 agonist loading (Lynn et al., 2015).

This detailed molecular and cellular insight confirms multiple previous observations highlighting the importance of formulation and physicochemical form of vaccine adjuvants for maximising immunostimulatory activity. For example, an increase in potency following liposomal formulation on muramyl dipeptide was observed as far back as 1984 (Sone et al., 1984) the immunostimulatory activity of which has been more recently attributed to nucleotide-binding oligomerization domain-like receptors (NOD) activation. Likewise, the *in vivo* adjuvant activity of TLR2 stimulatory lipopeptides was found to be strongly dependent on physical form, with particulate components primarily responsible for vaccine activity *in vivo* (Tsunoda et al., 1999). The physical form and self-assembly properties of TLR2 stimulatory lipopeptides have recently been studied in much greater detail, revealing that aggregation into micellular or fibrillar structures, has a profound effect on biological activity (Castelletto et al., 2016; Hamley et al., 2014).

The most intensely studied synthetic immunostimulatory small molecule PRR agonists target the sensors of viral RNA TLR7 and TLR8 (Heil et al., 2003; Hemmi et al., 2002; Takeda and Akira, 2004). Indeed, the imidazoquinoline Imiquimod was clinically approved in 1997, 5 years before its ability to trigger TLR7 was identified (Hemmi et al., 2002). Many studies have reported the relative potency of different families of small molecule agonists for TLR7 and TLR8, and while different cellular assays and endpoints are used, it is still informative to compare their potency which varies widely. To date, the most potent reported were in a library of 2‑substituted 8‑hydroxyadenines some of which had minimum effective concentrations (MEC) as low as 1–10 nM (Hirota et al., 2002). The majority of compounds in contrast have reported activity in the 0.1–10 μM range including imidazoquinolines with a ED_50_ or MEC in μM level (Kurimoto et al., 2004). Several structure-activity relationship (SAR) studies have focused on identifying a suitable location for conjugation to protein antigens or macromolecules without reducing potency following conjugation (Chan et al., 2009; Wille-Reece et al., 2005a; Wu et al., 2007a). Interestingly, one of the most studied examples, UC-1V150, is by no means the most potent TLR7 molecule in its unconjugated form, with EC_50_ of around 500 nM (Wu et al., 2007a). This contrasts with structurally similar 8‑oxoadenine derivatives reported with EC50 as low as 5 nM (Czarniecki, 2008) and rationally designed compounds based on pharmacophore molecular docking studies as low as 0.5 nM have been reported (Yu et al., 2013).

The potency of UC-1V150 is significantly increased following conjugation to proteins such as serum albumin (Wu et al., 2007b) even taking into account that the conjugates typically have a drug substitution ratio of up to 5:1 meaning that stated molar concentrations underestimate the total quantity of TLR7-agonist small molecule drug by up to 5-fold. Similarly conjugation of UC1V150 to the anti-CD20 antibody rituximab demonstrates a similar increase in potency compared to the free unconjugated TLR7 agonist (Gadd et al., 2015). Imidazoquinoline compounds such as 3M-012 conjugated to HIV Gag P41 protein show a similar increase in potency over the unconjugated and the protein mixed with TLR7/8 agonist (Wille-Reece et al., 2005b). As well as protein/peptide conjugation of TLR agonists, other published examples of macromolecular conjugates include polymers such as PEG (Chan et al., 2011), phospholipids (Chan et al., 2009) and glycoconjugates (Donadei et al., 2016; Shinchi et al., 2015). These examples typically note an increase in potency compared to the unconjugated agonist or agonist mixed with the antigen/scaffold. Other TLR agonists have likewise been conjugated to macromolecules with modulation of activity (Yu et al., 2017). Furthermore, conjugation alters *in vivo* responses including altered duration and location of cytokine induction, and when humoral response was studied modification of the antibody titre and ratio of immunoglobulin G (IgG) subtypes (Donadei et al., 2016; Shinchi et al., 2015; Smirnov et al., 2011). Further benefits of conjugation were also observed beyond simply increasing potency; for example conjugated or unconjugated TLR7 agonist were both able to induce dendritic cell (DC) maturation, but cross presentation was increased when TLR7 agonist was conjugated directly to antigen (Oh et al., 2011; Oh and Kedl, 2010).

A lipid modified member of the imidazoquinoline family TLR7 agonist was shown to be a potent vaccine adjuvant *in vivo*, but the *in vitro* immunostimulatory activity of this compound was not reported (Smirnov et al., 2011). However, the *in vitro* activity of the unconjugated agonist was reported in the low μM range similar for the structurally related R848 compound. In contrast to the highly potent UC-1V150 conjugated to the phospholipid DOPE that had EC_50_ of approximately 50 nM *in vitro* when analysing IL12p40 concentrations, compared to the unconjugated agonist of aproximatly 1 μM (Chan et al., 2009). Separately, immunostimulatory liposomal formulations have been studied extensively for vaccine formulation as an antigen delivery and/or depot system. While the majority of liposome-forming lipids lack intrinsic immunostimulatory activity, addition of the glycolipid trehalose‑6,6‑dibehenate (TDB) provides direct immunostimulatory activity to liposomes by triggering the PRR minkle *via* Syk/CARD9 (Matsunaga and Moody, 2009; Vangala et al., 2006; Yamasaki et al., 2008).

Here, we studied the influence of the physical form of lipid conjugated TLR7 agonist on immunostimulatory potency. We specifically focussed on phospholipid-conjugated forms of 2-substituted 8‑hydroxyadenines which are potent TLR7 agonists (Chan et al., 2009). The physical form of this conjugate and its influence on biological activity has not previously been reported, and we therefore describe here how either nanoparticles formed by dilution from solvent, or a liposomal formulation, are required for this conjugate to be active *in vitro* and provide further physical characterisation by SAXS.

## 2 Material and methods

### 2.1 Materials

RPMI 1640 supplemented with Glutamax & HEPES & Phenol Red, performance plus heat inactivated foetal bovine serum, phosphate buffered saline and 2‑mercaptoethanol were from Life Technologies (Paisley, UK). ExtrAvidin alkaline phosphatase conjugate, Sigmafast pNPP, PBS tablets, sodium azide and EDTA (0.5 M) were from Sigma Aldrich (Gillingham, UK). The IL-12p40 capture antibody C 15.6 and biotinylated IL-12p40/70 detection antibody C17.8 were from eBioscience (Hatfield, UK). Nunc® Maxisorb 96 well ELISA plates were from Thermo Fisher scientific (Paisley, UK). Reagents for chemical synthesis were purchased from Sigma Aldrich (Gliiingham, UK) or Fluorochem (Hadfield, UK) and used without further purification.

### 2.2 Synthesis

**Fig. 1:**
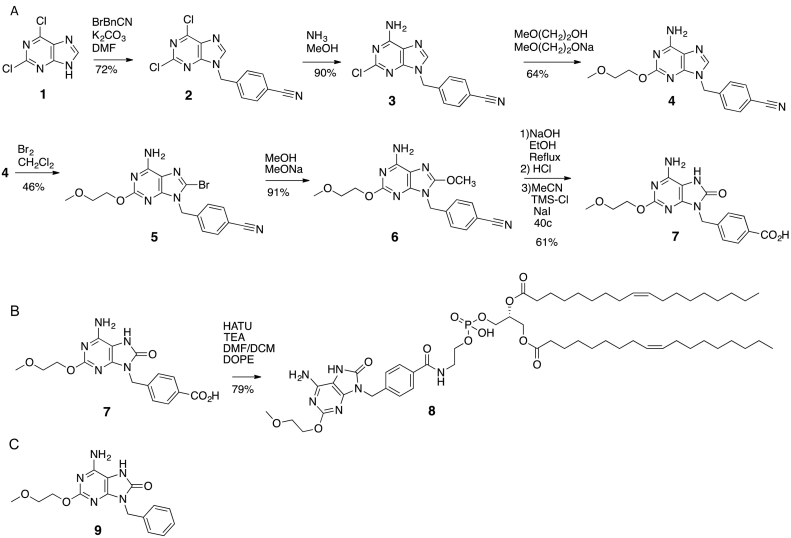
Synthesis scheme of carboxyl modified TLR7 agonist (A). Synthesis of DOPE-TLR7a agonist conjugate by conjugation of carboxy modified TLR7 agonist to phospholipid DOPE (B) and diagram of TLR7 agonist lacking a conjugation moiety (C).

TLR7 agonist with carboxyl site on the para-benzyl group for conjugation (**7**, Fig. 1) and DOPE-TLR7 agonist conjugate (**8**, Fig. 1) were synthesised according to previous methods (Wu et al., 2007b) and obtained in good yields as white crystalline powders. A similar TLR7 agonist without the carboxyl conjugation site on the para-benzyl group (**9**, Fig. 1) was also synthesised for activity comparison. Full synthetic methodology and comprehensive characterisation data is provided in Supplementary information, including a crystal structure for **9** (Supplementary information).

4‑{[6‑Amino‑2‑(2‑methoxyethoxy)‑8‑oxo‑7*H*‑purin‑9(8*H*)‑yl]methyl}benzoic Acid (**7**, Fig. 1)

^1^H NMR (DMSO‑*d*_6_) *δ* 12.94 (1H, s, ArC(O)OH), 10.03 (1H, s, NHC(O)N), 7.89 (2H, d *J* = 8.2, 2x *o*-ArH), 7.39 (2H, d, *J* = 8.2, 2x *m*-ArH), 6.51 (2H, s, NH_2_), 4.94 (2H, s, ArCH_2_N), 4.25 (2H, t, *J* = 4.2, OCH_2_CH_2_OCH_3_), 3.57 (2H, t, *J* = 4.2, OCH_2_CH_2_OCH_3_), 3.26 (3H, s, OCH_2_CH_2_OCH_3_). ^13^C NMR (DMSO‑*d*_6_) δ167.00, 159.83, 152.17, 149.08, 147.75, 141.98, 129.83, 129.55, 127.39, 98.34, 70.15, 65.26, 58.01, 42.12. HRMS calculated for C_16_H_18_O_5_N_5_^+^ (MH^+^) 360.1302 found 360.1302.

IR (*ν*, cm^−1^): 3351, 3165, 2956 (Broad), 1703, 1630, 1603

(2*R*)‑3‑(((2‑(4‑((6‑amino‑2‑(2‑methoxyethoxy)‑8‑oxo‑7,8‑dihydro‑9H‑purin‑9‑yl)methyl)benzamido)ethoxy)(hydroxy)phosphoryl)oxy)propane‑1,2‑diyl dioleate (**8**)

^1^H NMR (DMSO‑*d*_6_, 700 MHz, 45 °C): *δ* 10.35 (1H, s, NHC(O)N), 9.97 (1H, bs, ArC(O)NHCH_2_CH_2_), 9.37 (1H, bs, PO_3_OH), 7.86 (2H, d, *J* 8.2, 2x *o*-ArH), 7.34 (2H, d, *J* 8.2, 2x *m*-ArH), 6.60 (2H, bs, NH_2_), 5.31 (4H, m, 2x CH_2_CHCHCH_2_), 5.09 (1H, m, POCH_2_CHCH_2_O), 4.89 (2H, s, NCH_2_Ar), 4.29 (1H, dd, *J* 3.1, 12.0, C(O)NHCH_2_CH_2_OPO_3_) 4.25 (2H, m, OCH_2_CH_2_OCH_3_), 4.09 (1H, m, C(O)NHCH_2_CH_2_OPO_3_), 3.84 (2H, bs, POCH_2_CHCH_2_O), 3.81 (2H, bs, POCH_2_CHCH_2_O) 3.57 (2H, m, OCH_2_CH_2_OCH_3_), 3.26 (3H, s, OCH_2_CH_2_OCH_3_), 3.17, (1H, d, *J* 5.1), 2.24 (4H, m, 2x OC(O)CH_2_CH_2_), 1.95 (8H, m, 2x CH_2_CH_2_CHCHCH_2_CH_2_), 1.48 (4H, m, 2x OC(O)CH_2_CH_2_), 1.23 (40H, m, Lipid tail CH_2_), 0.85 (6H, m, 2x CH_2_CH_2_CH_3_).

^13^C NMR (DMSO‑*d*_6_, 175 MHz) *δ* 173.13, 172.88, 160.43, 152.74, 149.62, 148.45, 140.74, 134.04, 130.22, 128.01, 127.9, 99.00, 70.82, 62.93, 58.66, 46.04, 42.69, 40.59, 34.15, 33.97, 31.88, 29.70, 29.69, 29.44, 29.30, 29.23, 29.19, 29.12, 29.10, 29.03, 28.99, 19.16, 14.53.

HRMS calculated for C_57_H_94_N_6_O_12_P^+^ (MH^+^) 1085.6662 found 1085.6677

IR (ν, cm^−1^): 3438, 3352, 3193, 2927, 2856, 1742, 1708, 1674, 1638, 1617.

### 2.3 Endotoxin testing

Endotoxin is a component of bacterial cell walls with potent inflammatory response through TLR4 and is also a common laboratory contaminant. Synthesis and conjugation was completed in sterile environment and only endotoxin free reagents used. To confirm the absence of endotoxin contamination, a LAL assay was used to quantify the amount of soluble endotoxin in all reagents. Endotoxin content of sample was analysed by a Hycult biotech (Uden, Netherlands): LAL Chromogenic Endpoint Assay Endotoxin quantification kit and was performed according to manufacturer's instruction, unless otherwise stated all compounds tested were below the limit of detection of 0.08 EU/mL.

### 2.4 Formulation of nanoparticles and liposomes

#### 2.4.1 Nanoparticle formulation of DOPE-TLR7a agonist conjugate

Weighed amounts of DOPE-TLR7 agonist conjugates (typically 1 mg, 0.9 μmol) were pre-solubilized with DMSO (50 μL) before diluting with H_2_O to form a stock solution at 150 μM. Samples were then diluted in ether H_2_O or complete cell culture media (RPMI 1640, 10% FBS, 50 μM 2-ME) from the stock solution. Solutions of DOPE-TLR7 agonist conjugates were tested at concentrations relevant for *in vitro* stimulation of RAW 264.7 macrophages (1–1000 nM). Unconjugated TLR7a or DOPE-TLR7a conjugates stored as either dry powder or in complete media as nanoparticle formulations showed no difference in activity when stored at −20 °C over a period of 6 months. Although not formally evaluated, we have no evidence to suggest any instability of these conjugates upon storage.

#### 2.4.2 Production of liposomes containing TDB and DOPE-TLR7 agonist conjugates

Multilamellar vesicles (MLV) were synthesised by using the original lipid-film hydration method (Bangham et al., 1965). MLV were selected without further sonication or homogenization because previous studies had shown that liposomal size had relatively little impact on immunostimulatory potency (Henriksen-Lacey et al., 2011). DDA, TDB and DOPE-TLR7 agonist conjugate were dissolved in chloroform/methanol (9:1 by volume) to give 10 mg/mL stock solutions. DDA was used as the core constituent of MLVs and used at 60 w/v% when TDB was present or 80 w/v% when no TDB was incorporated. DOPE was used at 20 w/v% and substituted accordingly with DOPE-TLR7 agonist. The organic solvent was removed by rotary evaporation, followed by flushing with N_2_ to form a thin lipid film on the bottom of a round-bottomed flask. The lipid film was hydrated in 10 mM Tris-buffer at pH 7.4 by heating for 20 min at 60 °C. The phase transition temperature (T_m_) of DDA is 47 °C. This general method to formulate MLVs was used to produce a variety of liposomal formulations as listed in Table 1. MLV formulations were stable for a period of up to 2 weeks when stored at 4 °C, at which point aggregation and was evident and a reduction in activity was observed.

**Table 1:** Composition of MLVs containing DOPE-TLR7a conjugate, TDB and DOPE.

MLV composition	TDB w%/v% (μM)	DOPE-TLR7a agonist w%/v% (μM)	DOPE w%/v% (μM)
0% DOPE-TLR7a − TDB	0 (0)	0 (0)	20 (336)
0% DOPE-TLR7a + TDB	20 (250)	0 (0)	20 (336)
1% DOPE-TLR7a − TDB	0 (0)	1 (11.5)	19 (320)
20% DOPE-TLR7a − TDB	0 (0)	20 (230)	0 (0)
20% DOPE-TLR7a + TDB	20 (250)	20 (230)	0 (0)

To clarify the final concentrations of immuno-modulatory components in each preparation and the calculations involved to determine the concentration of compounds used *in vitro* are described in more detail as follows. To produce MLVs with 20 w%/v% of TLR7a-DOPE conjugate: 125 μL of DDA (10 mg/mL) was transferred to a round bottom flask with the addition of 25 μL of DOPE-TLR7a conjugate (10 mg/mL). Following the above procedure, the lipid cake was then resuspended in 1 mL of Tris-buffer to give a final following concentration of compounds: DDA = 1.25 mg/mL or 1.98 mM, DOPE-TLR7a = 0.25 mg/mL or 230 μM, TDB = 0.25 mg/mL or 250 μM. MLV were then diluted into complete culture medium to the desired concentration for *in vitro* stimulation assays. When both TLR7a-DOPE conjugate and TDB were present in MLVs, the concentration of DOPE-TLR7a was used to determine dosing during *in vitro* stimulations assays.

### 2.5 Particle size analysis using Nanosight Tracking Analysis (NTA) and Dynamic Light Scattering (DLS)

Samples were prepared in 1 mL volume using milliQ ultrapure water for NTA analysis, filtered through a 0.45 μm syringe filter immediately prior to use. NTA was performed on a LM10 system with LM14 top plate and 533 nm laser (Malvern, UK). Flow rate was determined by software and set to 30 AU. Videos were recorded 5 time for each sample, 60 s each, samples were repeated 3 times at a controlled temperature of 25 °C. A 200 nm particle standard was used as a calibration run diluted 1:1000 in miliQ water. For samples analysed in complete media, detection threshold was increased to 6 to reduce autofluorescece noise. All analysis was performed using NTA software v3.1 (Malvern, UK).

Liposomes were characterized using DLS measured used a Zetasizer Nano-ZS (Malvern, UK). Samples were diluted into ultrapure water to appropriate concentrations in low volume plastic cuvettes (Fisher Scientific). A dispersant refractive index of 1.33 and material refractive index of 1.45 was used for all samples. Individual measurements were carried out for 10 s per run, with 12 runs per reading, repeated in triplicate. This was repeated for three separate samples at 25 °C. ξ-Potential values were also measured; using DTS-1061 folded capillary tube cuvettes (Malvern, UK). Samples were diluted 1:100 in ultrapure water to the same concentration used in sizing experiments. Samples were measured using 20 sub-runs per reading, repeating 3 times for each sample. Each sample was measured three times and the results were processed using the Smoluchowski model (Fκa = 1.50).

### 2.6 SAXS characterisation of DOPE-TLR7a conjugates

#### 2.6.1 Sample preparation for Small Angle X-ray Scattering (SAXS)

An aliquot of DOPE-TLR7a conjugate was first dissolved in DMSO then further diluted in TRIS buffered saline (TBS; 0.05 M Tris, 0.15 M NaCl, pH 7.6) to give the corresponding concentrations used for SAXS experiments. 1 wt% DOPE-TLR7a in 0.02 vol DMSO/vol TBS was diluted with TBS to give (for example) 0.25 wt% DOPE-TLR7a in 0.005 vol DMSO/vol TBS.

#### 2.6.2 Small Angle X-ray Scattering (SAXS)

Synchrotron SAXS experiments on solutions were performed using a BioSAXS robots at beamline BM29 (ESRF, France). Solutions were loaded into the 96 well plate of an EMBL BioSAXS robot, and then injected *via* an automated sample exchanger into a quartz capillary (1.8 mm internal diameter) in the X-ray beam. The quartz capillary was enclosed in a vacuum chamber, in order to avoid parasitic scattering. After the sample was injected in the capillary and reached the X-ray beam, the flow was stopped during the SAXS data acquisition. BM29 operated with an X-ray wavelength λ = 1.03 Å (12 keV). The images were captured using a PILATUS 1M detector, while data processing was performed using dedicated beamline software ISPYB (BM29).

### 2.7 *In vitro* immunostimulatory activity with RAW264.7 macrophage cell line

Raw 264.7 macrophages were subcultured according to ATCC methods: Complete media contained RPMI 1640 Glutamax® (10% FBS, 2‑Mercaptoetanol 0.05 mM). Cells were cultured at 37 °C 5% CO_2_ in T-75 flasks until approximately 80% confluency. Cells were then detached using a solution of EDTA in PBS (1 mM, 10 mL) per T-75. Cell suspensions were then centrifuged at 200 ×*g* for 5 min. The supernatant was removed then the pellet re-suspended in complete media (5 mL). Cells were then re-suspended in fresh culture media at 2 × 10^5^/mL.

Stimulation assays were performed in tissue culture grade 96 well plates. Test compounds were diluted serially 1:2 (100 μL final volume) in complete media (RPMI 1640, 10% FBS, 0.05 mM 2-ME) then 2 × 10^6^ cells/mL freshly passaged cells were added in 100 μL to give a final volume of 200 μL. Plates were incubated for 24 h before the analysis of supernatants for pro-inflammatory cytokines. IL-12p40 concentrations in supernatants of RAW 264.7 stimulation assays were determined by sandwich ELISA. Briefly, Nunc Maxisorb 96 well ELISA plates were coated overnight with IL-12p40 capture antibody (C15.6) at 2 μg/mL in carbonate buffer (pH 9.6, 50 μL per well), followed by 2 h with 200 μL per well block buffer (PBS, 2.5 v/v% FBS, 0.02 w/v% NaN3). 100 μL per well biotinylated detection antibody (C17.8) at 1 μg/mL in block buffer was added. Stimulated RAW 264.7 supernatants were diluted 1:7.5 in block buffer, and compared to 1:2 dilution of recombinant IL-12p40 standards with a top concentration of 10 ng/mL in block buffer, with 150 μL sample per well. ExtrAvidin alkaline phosphatase conjugate was prepared 3:10,000 dilution in PBS.

## 3 Results

### 3.1 *In vitro* immunostimulatory activity of lipid conjugate nanoparticles

**Fig. 2:**
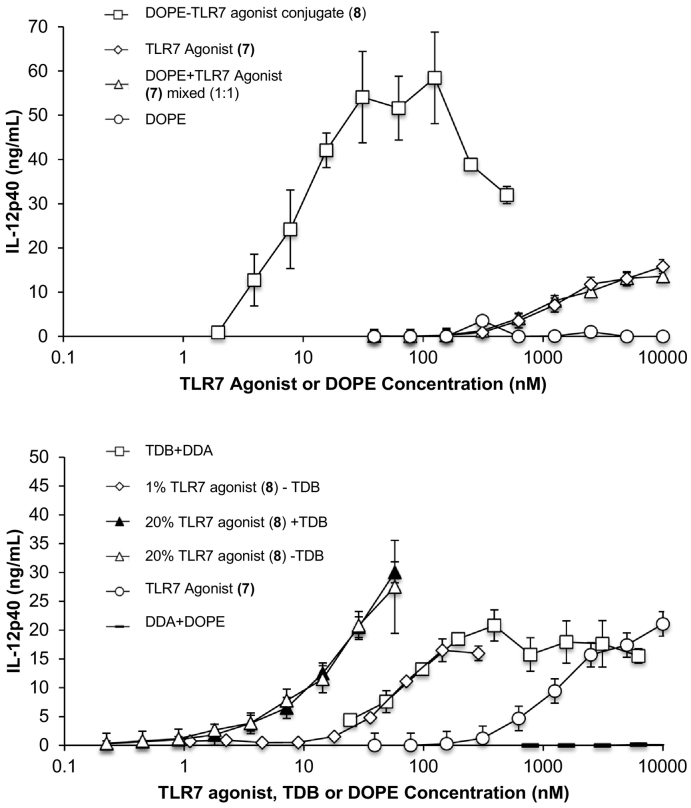
IL-12p40 concentration in supernantants of RAW 264.7 macrophages cultures stimulated with Unconjugate TLR7 agonist, Unconjugated DOPE, TLR7 agonist mixed with DOPE 1:1 and DOPE-TLR7a conjugate (A). IL-12p40 concentration in supernatants of RAW 264.7 macrophages cultures after stimulation with MLV containing DDA + TDB, DDA + DOPE, DDA + 1% (w/v) DOPE-TLR7a conjugate, DDA + 20% (w/v) DOPE-TLR7a conjugate, DDA + TDB 20% (w/v) + 20% (w/v)DOPE-TLR7a conjugate and TLR7 agonist (B). n = 3, error bars show standard deviation.

The TLR7 agonist UC-1V150 was conjugated to the phospholipid DOPE as previously described and noted for their remarkable potency compared to the unconjugated TLR7 agonist (Chan et al., 2009). We discovered that pre-dissolving of the DOPE-TLR7a agonist conjugate in DMSO was required to detect any activity with *in vitro* stimulation (Table 2 and Fig. 2). We hypothesised that DOPE-TLR7a would be likely to form micelles or nanoparticles in aqueous suspension following dilution from DMSO solution. To investigate the potency of alternative formulations of the lipid-conjugated TLR7 agonist we added the conjugate to DOPE:DDA liposomes and found that this likewise increased potency by >10-fold compared to unconjugated TLR7 agonist (Fig. 2B and Table 2, Table 3).

**Table 2:** Immunomodulatory properties of TLR7 agonists, conjugates and liposomes.

	Sample	EC50 nM	MEC nM
Conjugates	Unconjugated TLR7 Agonist (**8**)	483 ± 25	74
	Unmodified TLR7 Agonist (**9**)	620 ± 19	190
	DOPE-TLR7a + DMSO	9 ± 1	<1
	DOPE-TLR7a − DMSO	No activity (>2000 nM)	No activity (>2000 nM)
Liposomes	MLV 1% DOPE-TLR7a	45 ± 23	6
	MLV 20% DOPE-TLR7a	34 ± 20	<2
	MLV 20% DOPE-TLR7a + TDB	33 ± 19	<2
	MLV + TDB	223 ± 173	<24
	MLV no immunostimulatory agent	>200,000	>200,000

**Table 3:** *Z*-Average, PDI and Zeta potentials of MLVs determined by DLS.

LIPOSOME composition	Z-average size (nm)	PDI	Zeta potential (mV)
0% DOPE-TLR7a − TDB	636	0.45	59 ± 9
0% DOPE-TLR7a + TDB	679	0.18	76 ± 20
1% DOPE-TLR7a − TDB	424	0.80	67 ± 12
20% DOPE-TLR7a − TDB	526	0.34	65 ± 11
20% DOPE-TLR7a + TDB	616	0.37	60 ± 20

Liposomes were analysed by DLS and found to have similar characteristics to DDA:TDB MLVs. The incorporation of TLR7-agonist conjugated DOPE had little effect on size distribution (Table 3). Like DMSO-dissolved/diluted nanoparticles, liposomes containing TLR7 agonist showed potent *in vitro* immunostimulatory activity with an EC50 of around 9 nM (Table 2, Fig. 2B). Interestingly, when we changed the liposomal composition by reducing the TLR7 content, a significant reduction in activity was observed and found that there was no additive or synergistic increase in activity when TLR7 agonist was combined with the Minkle/Syk/CARD9 activating TDB. This was in line with previous studies where TDB was shown not to synergise with TLR3 and TLR9 agonists for vaccine potency in MLVs (Milicic et al., 2012).

### 3.2 High potency of lipid conjugated TLR7 agonist depends on nanoparticle formation or liposomal formulation

The poor water solubility of the lipid conjugated TLR7 agonist prevents direct addition of this compound to *in vitro* immunostimulatory assays, and it was therefore diluted in DMSO prior to dilution into culture. We hypothesised that DOPE-TLR7a would be likely to form micelles or nanoparticles in aqueous suspension following dissolution in DMSO and dilution into water or culture medium. We therefore used nanosight particle tracking analysis (NTA) to determine the physical form of this preparation, and found nanoparticles with a mean diameter of 93 ± 1 nm when diluted into ultrapure water. Complete cell culture medium showed background particulate matter as expected given the high protein content and heterogenous composition of heat inactivated foetal bovine serum. A background fluorescence was also observed from phenol red in the culture medium, which increased noise and reduced sensitivity with control 100 nm latex particles. The increased viscosity of complete media over H_2_O was taken into account by software correction viscosity values at 25 °C for water = 0.89 cP and complete media = 0.96 cP. However, when diluted from DMSO into culture medium an increase in size of the DOPE-TLR7 agonist conjugate was observed comparing to ultrapure water. The increase from 93 ± 1 nm in water compared to 155 ± 5 nm in culture medium could be attributed to agglomeration of particles, or to incorporation of media components into micelles/nanoparticles. The reduction of particle frequency in DOPE-TLR7a conjugates - not mirrored by the 100 nm latex standard - also supported the possibility of agglomeration (Table 4).

**Table 4:** Particle frequency of DOPE-TLR7a conjugate or 100 nm latex standards in complete media or milliQ water determined by NTA.

Sample	Complete media counts per mL	H_2_O counts per mL
DOPE-TLR7a	3.6 × 10^8^	8 × 10^8^
100 nm latex	1.15 × 10^9^	1.2 × 10^9^
Blank	1 × 10^6^	5 × 10^5^

### 3.3 Structure analysis

To determine the physical form of the lipid conjugate in micellar/nanoparticulate form, 0.15, 0.4 and 1 wt% DOPE-TLR7a was prepared by dissolution/dilution and analysed by SAXS (Fig. 4). The SAXS scattering shows two distinct peaks, at spacings *d* = 2π /*q*_*o*_ = 68.7 and 33.9 Å (*q*_*o*_ = position of the maxima in the scattering curve) and at a positional ratio 1:2. These peaks suggest that DOPE-TLR7a is arranged in a lamellar order with a layer spacing of 68.7 Å. The SAXS data for 1 wt% DOPE-TLR7a was modelled according to the form factor for a bilayer structure, comprising three Gaussian functions to represent the electron density variation across the two headgroups and the dense lipid core (Pabst et al., 2000). The interaction between the bilayers was described by a modified Caillé structure factor (Caillé, 1972). The model and its application to lipid-based bilayer structures have been described in detail in our previous papers (Castelletto et al., 2013, Castelletto et al., 2012). The fitting was implemented using the software SASfit (Breßler et al., 2015). Fig. 4b shows the fitting of the SAXS data for 1 wt% DOPE-TLR7a using this model. The fitted parameters are listed in Table 5, and the organization of DOPE-TLR7a, according to data in Fig. 4 and the parameters in Table 5 is illustrated in Fig. 5.

**Table 5:** SAXS parameters extracted from the fittings of the experimental data shown in Fig. 4b.

*2z*_*H*_ [Å]	Δ_2*zH*_ [Å]	ρ_H_ [rel. units]	σ_H_ [Å]	ρ_C_ [rel. units]	σ_C_ [Å]	*N*	*d*_*o*_ [Å]	*η*	*N*_*diff*_
66	25.9	9.3e−4	5.7	1.2e−4	5.7	3.2	67	2.2	3.5

Key: Gaussian bilayer form factor: Gaussian half-width at half-maximum for polydispersity Δ_2*zH*_, inter-head group thicknesses 2*z*_*H*_, Gaussian half-width for outer layer surface σ_H_, electron density for headgroup ρ_H_, Gaussian half-width for inner layer σ_C_, relative electron density for inner layer ρ_C_. Caillé structure factor: diffuse background *N*_*diff*_; total number of layers *N*; layer thickness *d*_*o*_; Caillé parameter *η*.

The fitting of the SAXS data shows that DOPE-TLR7a molecules are organized in bilayers with a repeat distance of 77 ± 25 Å, such that the parameters listed in Table 5 are correlated in groups over ~3 bilayers. The repeat distance provided by the SAXS fitting is in good agreement with the lamellar parameter 68.7 Å measured from the SAXS curve for 0.14–1 wt% DOPE-TLR7a (Fig. 4a). In contrast, SAXS experiments measuring the structure of pure DOPE lamellar provide a layer spacing of 74.6 Å in water (Koltover, 1998).

## 4 Discussion

**Fig. 3:**
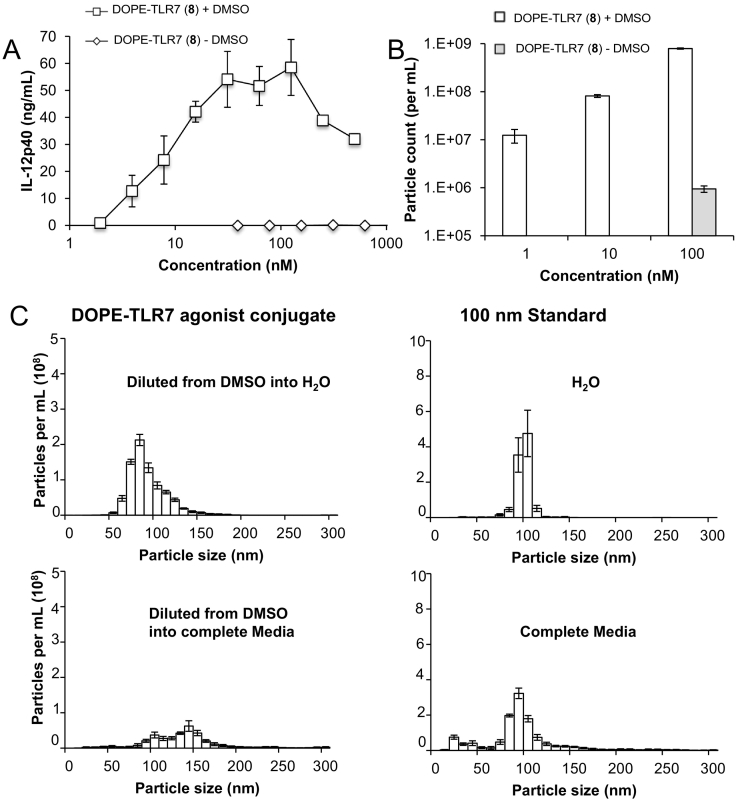
IL-12p40 concentration in supernatants of RAW 264.7 macrophages cultures after stimulation with DOPE-TLR7 agonist conjugate with or without pre-dissolving in DMSO (A). Particle frequency determined by NTA tracking analysis of DOPE-TLR7 agonist conjugate with or without pre-dissolving in DMSO (B). The effect of solvent on particle size of DOPE-TLR7 agonist conjugates pre-dissolved in DMSO, (top) H_2_O (bottom) complete media. In addition 100 nm latex standards were also run in the same solvent systems (C). n = 3–5 error bars show standard deviation.

Conjugation of TLR7a to the phospholipid DOPE has previously been shown to promote strong pro-inflammatory response *in vitro*, with a similar increase in potency to other TLR7/8 agonist conjugates to macromolecules such as polymers and peptides. However the inadvertent self-assembly of this compound by dissolving in DMSO prior to addition to culture, to overcome the poor aqueous solubility has not previously been investigated. Likewise, the potency of liposomes incorporating DOPE-TLR7a conjugates has not been reported. We show that attempts to directly disperse DOPE-TLR7a directly into aqueous solution shows no sign of particulates when analysed by light scattering techniques (Fig. 3B) and likewise no *in vitro* pro-inflammatory activity can be detected without dilution from DMSO solution or incorporation into liposomes (Table 2 and Fig. 3A). Separately dissolving the TLR7a agonist plus DOPE in DMSO before mixing and diluting in aqueous media showed no increase in the *in vitro* pro-inflammatory response, demonstrating incorporation *via* covalent coupling to lipid group is essential to influence potency (Fig. 2A). Similar activity was observed between TLR7 agonists without conjugation to the lipid group either with a functional group for conjugation, such as a carboxylic acid (**7**), and without functional group at the para-benzyl position (**9**), suggesting the alteration of activity is due to lipid conjugation not simply variation in groups on this para-benzyl position (Table 2). We were able to diffract a crystal of compound **9** to confirm for the first time the solid state structure of this important immunostimulatory compound (Supplementary information).

**Fig. 4:**
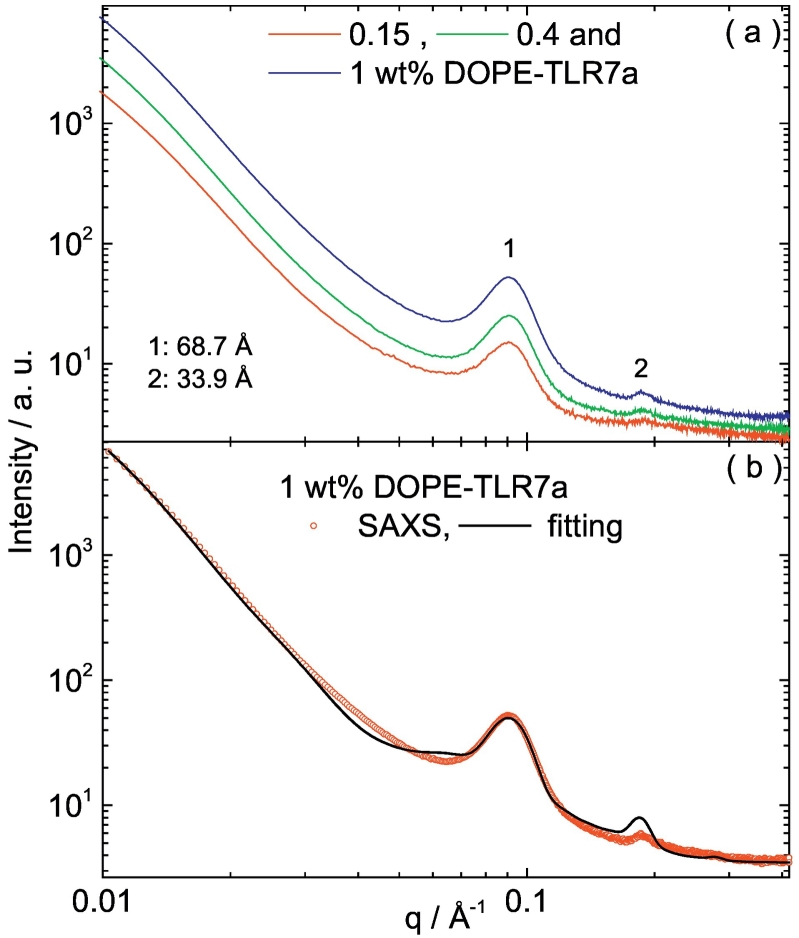
(a) SAXS data measured for samples containing 0.15, 0.4 and 1 wt% DOPE TLR7a. (b) SAXS data for 1 wt% DOPE-TLR7a fitted according to the form factor for a lipid bilayer with a Gaussian electronic density profile, as described in the text and fitted parameters in Table 5.

**Fig. 5:**
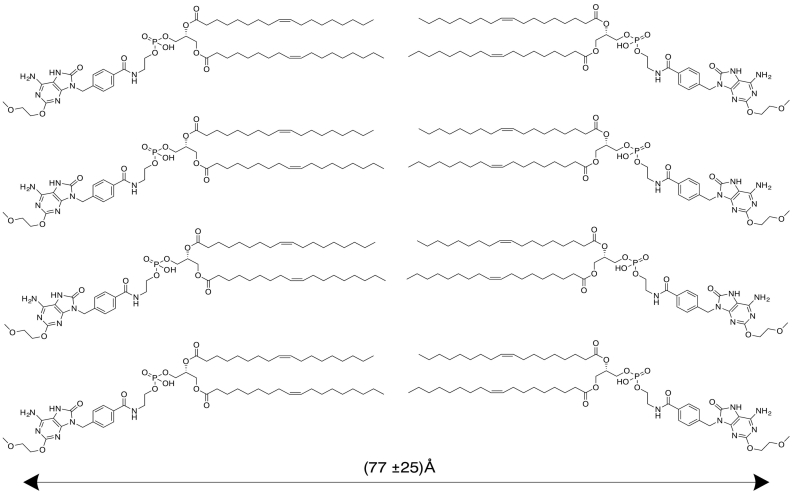
Lamellar order of DOPE-TLR7a molecules and repeat distance of the bilayer, according to the fitting of the SAXS data measured for 1 wt% DOPE-TLR7a (Fig. 4, Table 5).

Either using DMSO to dissolve the DOPE-TLR7a conjugate prior to dispersion by dilution in aqueous solvents, or using well-established lipid cake hydration method of forming MLVs showed a clear and large increase in the number of particles formed and also a strong pro-inflammatory response *in vitro* (Fig. 3). Complete culture media typically contains 10% FCS in addition to a large amount of amino acids, minerals and carbohydrates. Changes in ionic strength, counterions, protein concentration and pH can influence the formation, stability and aggregation of particles (Maeda et al., 1997; Palladino and Ragone, 2011). Changing the solvent from water to complete media influenced the average particle size and frequency of DOPE-TLR7a particles detected by light scattering (Fig. 3c and Table 4). The increase in particle size and reduction in particle frequency could be explained by the agglomeration of DOPE-TLR7a particles. Control 100 nm latex particles analysed in complete media displayed a broadening of particle size, the mean increasing from 100 nm to 120 nm but particle frequency remained constant (Table 4), suggesting that complete media may interact directly with DOPE-TLR7a affecting particle size and frequency.

TDB is not only an immunostimulatory glycolipid, but has also been reported to have a stabilising effect on cationic MLVs constructed from DDA and DOPE (Christensen et al., 2007). Using DLS to analyse MLV size distribution (Table 3) indicated that the inclusion of TDB did reduce the PDI of MLV formed from DDA, DOPE plus DOPE-TLR7a. The resulting MLV all showed a strong pro-inflammatory induction *in vitro*. Previous reports suggested that the immunostimulatory activity of TDB does not synergise with TLR 3 and 9 agonists,(Milicic et al., 2012) and we similarly found no evidence of synergistic effect of combining DOPE-TLR7a and TDB within liposomes in this simple *in vitro* system. Adding TDB to MLV containing 20% DOPE-TLR7a showed no increased pro-inflammatory activity (Fig. 3). Further study is now required to evaluate the impact on formulation on different cell types expressing TLR7, and molecular methods such as evaluation in knockout cells, or reporter cell lines exclusively expressing a panel of TLRs and other PRR, is now justified to understand if conjugation also affects receptor specificity, as well as potency, of these compounds. Initial experiments using a GFP inducible Nf-κB cell line lacking TLR7 known as GTPT3 (Bloor et al., 2008) showed no induced GFP expression over background when stimulated with unconjugated TLR7 agonist, DOPE-TLR7a conjugate or liposomes containing DOPE-TLR7a conjugate, but did show a dose dependant response to LPS (data not shown). This preliminary test suggests that DOPE-TLR7a conjugate in micellar or liposomal formulation remains specific for TLR7.

## 5 Conclusions

This study links the physical form of DOPE-TLR7a conjugates in aqueous dispersion to their biological activity *in vitro*, which has not been reported previously. Our data shows that the formation of TLR7 agonist containing nanoparticles distinctly modulates pro-inflammatory activity. This can easily be demonstrated by the aqueous dispersion of lipid conjugates from DMSO solution resulting in nanoparticle formation and high potency. Similarly, the formation of DOPE-TLR7a particles in the form of MLV liposomes significantly increased *in vitro* pro-inflammatory potency over the unconjugated TLR7a. SAXS data provided insight into the physical form of the conjugate after DMSO dissolution and aqueous dispersion, identifying a lamellar structure with repeat distance of 77 ± 25 Å, and with the modelled parameters listed in Table 5 correspond with groups of lamella in repeats with 3 bilayers. When liposomes containing DOPE-TLR7a conjugate were compared to previously characterized DDA:TDB liposomes, the most dramatic increase in potency was observed simply by the initial formulation into liposomes, rather than by combining multiple immunostimulatory agents. We demonstrated control over the DOPE-TLR7a content integrated in the liposomes and highlight the potential of liposomal formulation of this TLR7 agonist to achieve highly potent PRR activation. Formulations of particles containing TLR7a either as MLV or micelles *via* dissolution into DMSO prior to aqueous dilution are both equally potent inducers of IL-12p40. The most significant change in pro-inflammatory activity following lipid conjugation could be attributed to the formation of sub-micron particles. Overall, this study highlights the importance of physical form and formulation on activity of immunostimulatory compounds which trigger PRR.

## Author information

No competing financial interests have been declared.

## Appendix A Supplementary data

Supplementary material Image 1
