# Supplementary material for: High potency of lipid conjugated TLR7 agonist requires nanoparticulate or liposomal formulation
Source: Eur J Pharm Sci. 2018 Oct 15;123:268–76. doi: 10.1016/j.ejps.2018.07.048 (PMC6137072; doi:10.1016/j.ejps.2018.07.048)
Supplement: Supplementary file 1 — Supplementary material [file mmc1.pdf]

Supplementary Information

High Potency of Lipid Conjugated TLR7 Agonist Requires Nanoparticulate or Liposomal Formulation

Adam J. R. Gadd[1#], Valeria Castelletto[1], Elena Kabova[1], Kenneth Shankland[1], Yvonne Perrie[2], Ian Hamley[1], Alexander J. A. Cobb[3], F Greco[1], Alexander D. Edwards*[1]

[1] School of Chemistry Food and Pharmacy, University of Reading, Whiteknights, Reading, RG6 6AD, United Kingdom

[2] Strathclyde Institute of Pharmacy and Biomedical Sciences, University of Strathclyde, Glasgow, G1 1XQ, United Kingdom

[3] Department of Chemistry, King's College London, London, SE1 1DB, United Kingdom

* Corresponding author: a.d.edwards@reading.ac.uk +44 118 3784253

[#] Current address: School of Biological Sciences, University of Reading, Whiteknights, Reading, RG6 6AD, United Kingdom

Experimental

NMR Data: ^{1}H NMR spectra were recorded using a Bruker Avance III 400 (400 MHz) spectrometer or Bruker Avance III 700 (700 MHz). Chemical shifts (δ) are quoted in parts per million using the following abbreviations: singlet; s, broad singlet; bs, doublet; d, double doublet; dd, triplet; t, triple triplet; tt, quartet; q, multiplet; m. ^{13}C spectra were recorded using a Bruker Avance III 400 (100 MHz) or a Bruker Avance III 700 (175 MHz) spectrometer. All spectra were collected at 400 MHz or 100 MHz for ^{1}H or ^{13}C respectively unless otherwise stated.
Mass Spec Data: High resolution mass spectra were recorded on a Thermoscientific LQT Orbitrap XL under electron spray ionization conditions (ESI).
IR spectroscopy: Infrared spectra were recorded on a Thermo Scientific Nicolet iS5 FT-IR spectrometer using iD5 ATR accessory with absorptions quoted in wavenumbers (cm^{-1}).
X-ray Single crystal structure: Single crystal data were collected at 151K with copper radiation using an Oxford Diffraction Gemini diffractometer. Full details of the crystal structure are contained in the CIF which has been deposited (deposition number 1825910) with the Cambridge Crystallographic Data Centre *.
* These data can be obtained free of charge via www.ccdc.cam.ac.uk/data_request/cif, or by emailing data_request@ccdc.cam.ac.uk, or by contacting The Cambridge Crystallographic Data Centre, 12, Union Road, Cambridge CB2 1EZ, UK

Synthesis and Characterisation Data

4-(2,6-dichloropurin-9-ylmethyl)benzonitrile (**2**)

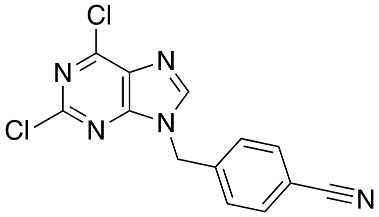

Figure S1. Diagram of 4-(2,6-dichloropurin-9-ylmethyl)benzonitrile (**2**)

Compound **1** (16 mmol, 3 g) was dissolved in DMF (50 mL) and K_2CO_3 (50 mmol, 7 g) was added and stirred for 30 mins. 4-(bromomethyl)benzonitrile (22 mmol, 4.31 g) was then added to the reaction mixture and stirred at RT until the reaction was complete, determined by TLC. The reaction mixture was filtered then added to deionised H_20 (1600 mL) and stirred overnight or until a yellow precipitate formed. The solution was then split (2x 800 mL) and each part extracted with EtOAc (2x 100 mL). The organic layer was then concentrated and the residue was subject to flash silica gel chromatography using 1:2:7 EtOAC, DMK, hexane. ^{1}H NMR (DMSO-*d*$_6$) δ 8.86 (1H, s, NC<u>H</u>N), 7.85 (2H, d, J= 8.4, 2x *m*-ArH), 7.51 (2H, d, J = 8.4, 2x *o*-ArH), 5.62 (2H, s, ArC<u>H</u>$_2$N). ^{13}C NMR (DMSO-*d*$_6$) δ153.5, 151.2, 149.8, 148.4, 141.1, 132.7, 130.6, 128.4, 118.5, 110.8, 46.6, HRMS calculated for $C_{13}H_8Cl_2N_5^+$ (MH^+) 304.0151 found 304.0155. m.p 201-202°c

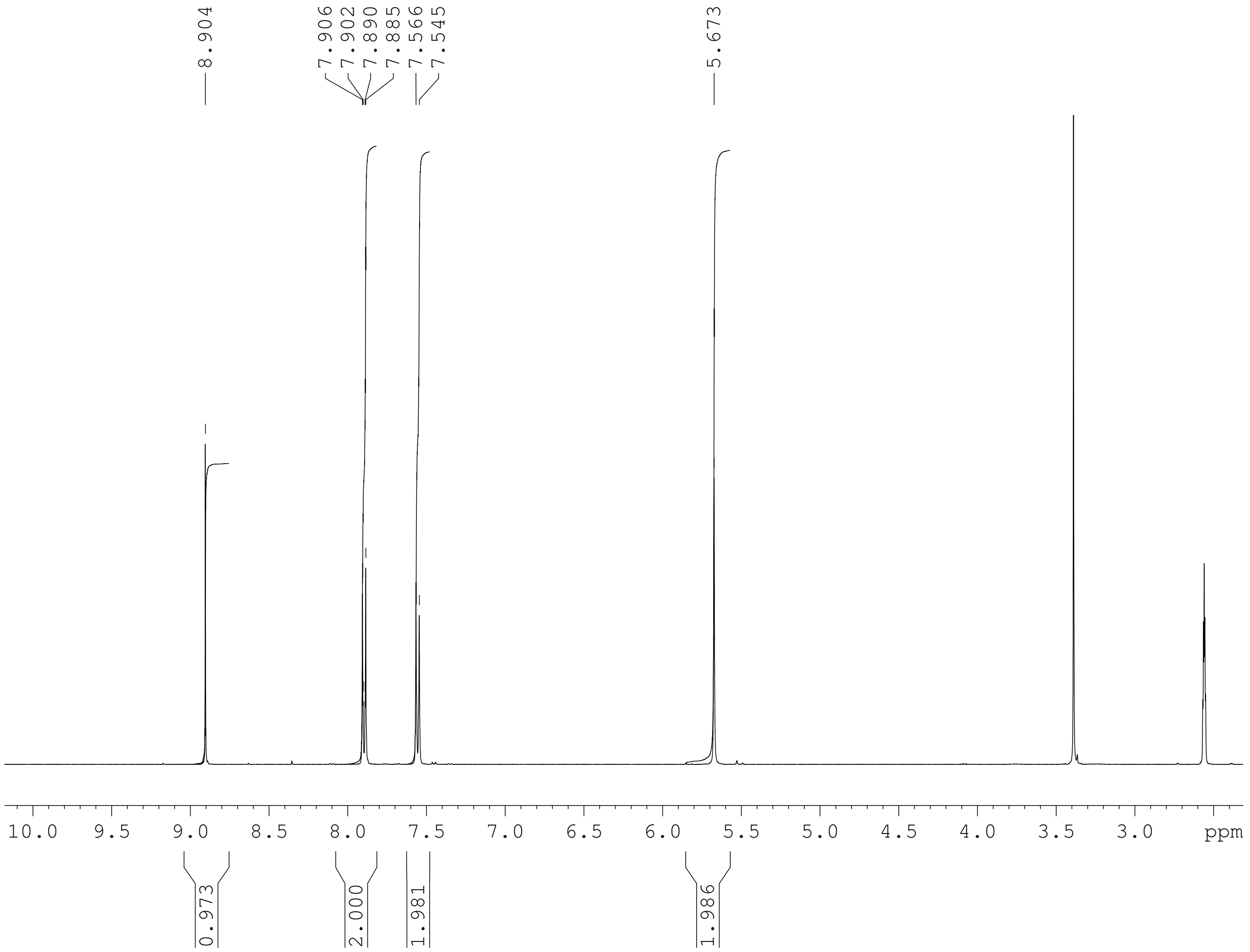

Figure S2. ^{1}H NMR of 4-(2,6-dichloropurin-9-ylmethyl)benzonitrile (**2**)

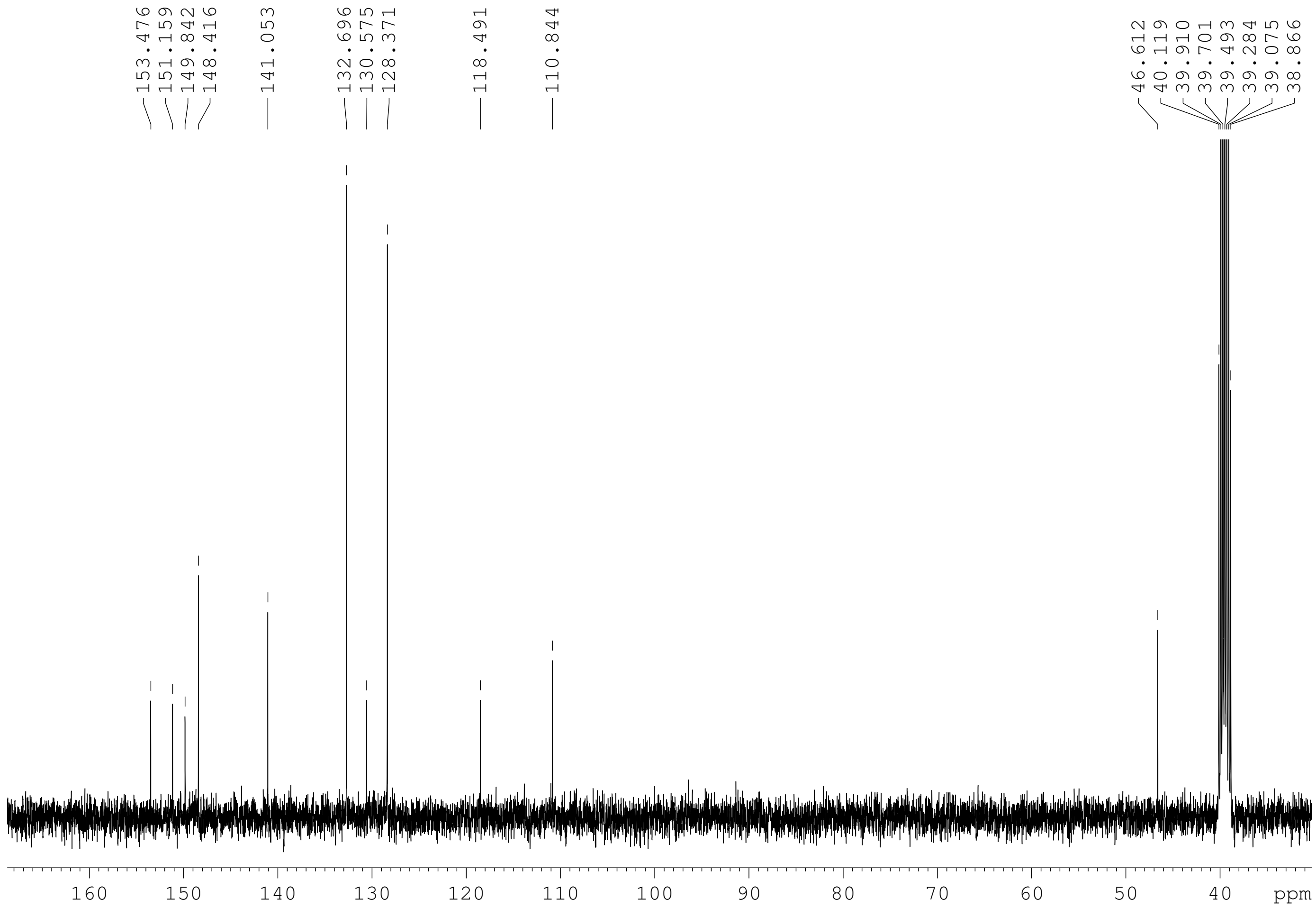

Figure S3. ^{13}C NMR of 4-(2,6-dichloropurin-9-ylmethyl)benzonitrile (**2**)

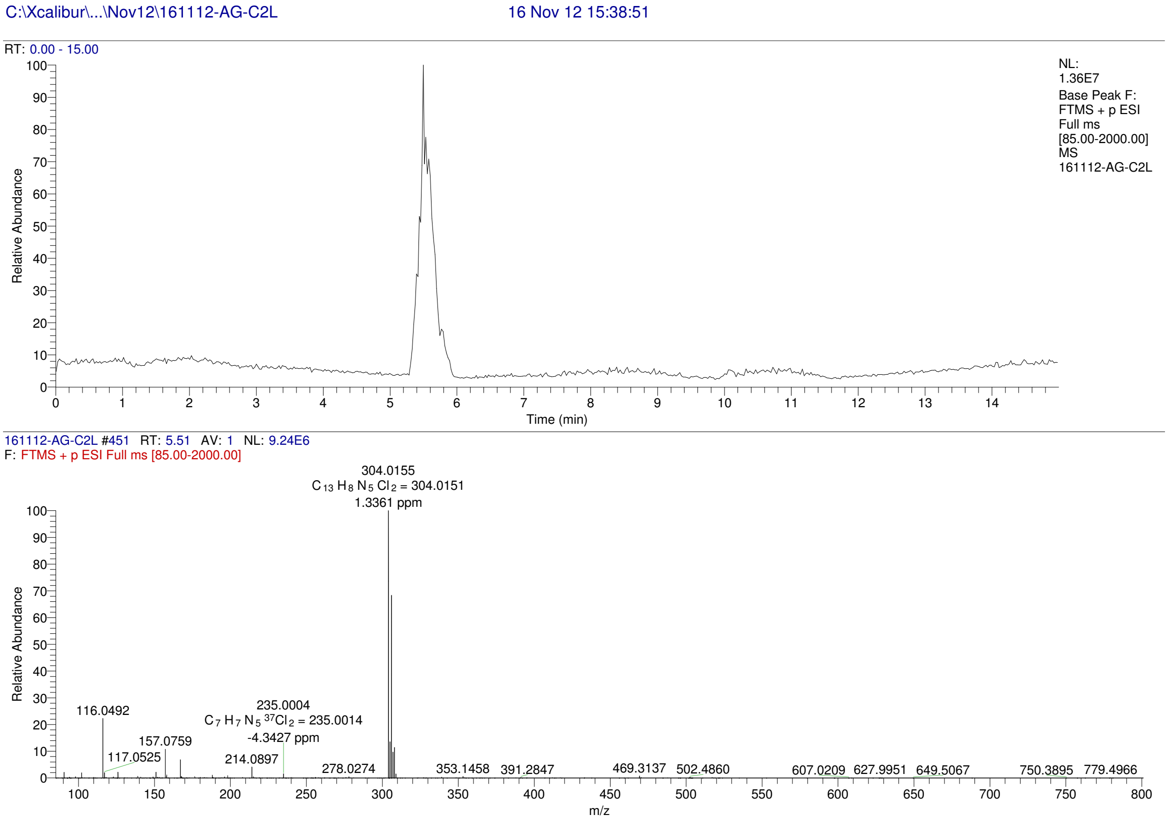

Figure S4. HRMS of 4-(2,6-dichloropurin-9-ylmethyl)benzonitrile (**2**)

4-(6-amino-2-chloropurin-9-ylmethylbenzonitrile (**3**)

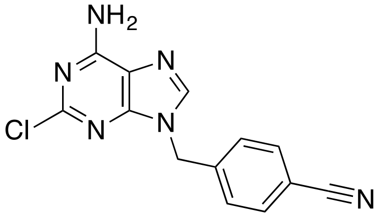

Figure S5. Diagram of 4-(6-amino-2-chloropurin-9-ylmethylbenzonitrile (**3**)

Compound **2** (3 mmol, 0.91 g) and methanolic ammonia (20 mL, 7 M) were added to a sealed pressure reaction vessel. The reaction mixture was then heated for 12 h at 60 °C. The mixture was cooled over ice and the solid product was filtered off. ^{1}H NMR (DMSO-d_6) δ 8.28 (1H, s, NCHN) 7.83 (2H, d, J = 8.4, 2x *m*-ArH), 7.81 (2H, s, NH_2), 7.42 (2H, d, J = 8.4, 2x *o*-ArH), 5.46 (2H, s, ArCH_2N). ^{13}C NMR (DMSO-d_6) δ160.9, 156.8, 153.1, 151.6, 150.5, 144.5, 142.2, 141.7, 141.5, 132.7, 128.3, 118.5, 117.7, 110.6, 45.6. HRMS calculated for $C_{13}H_{10}ClN_6^+$ (MH^+) 285.0650 found 285.0651. mp 287-288°c

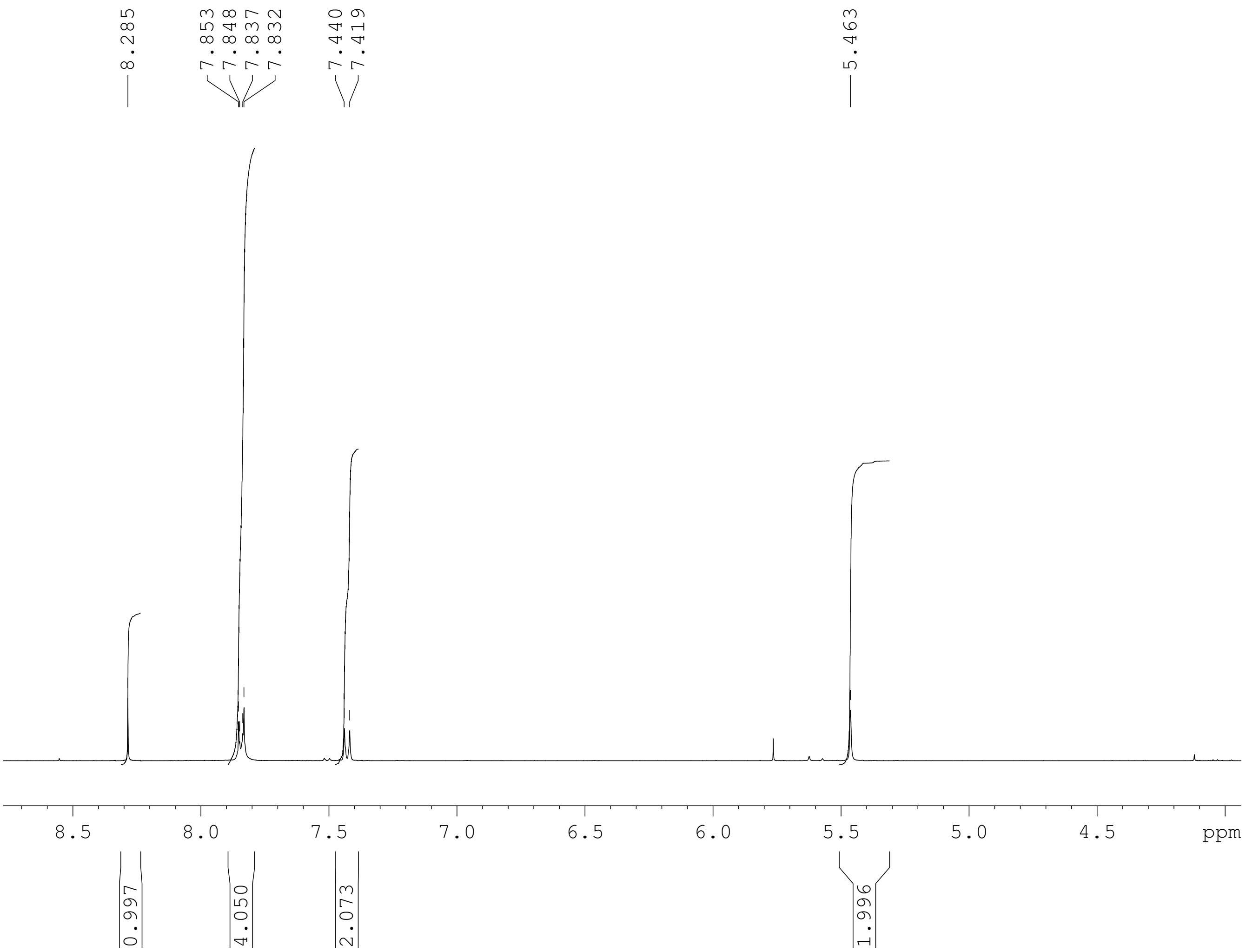

Figure S6. ^{1}H NMR of 4-(6-amino-2-chloropurin-9-ylmethylbenzonitrile (**3**)

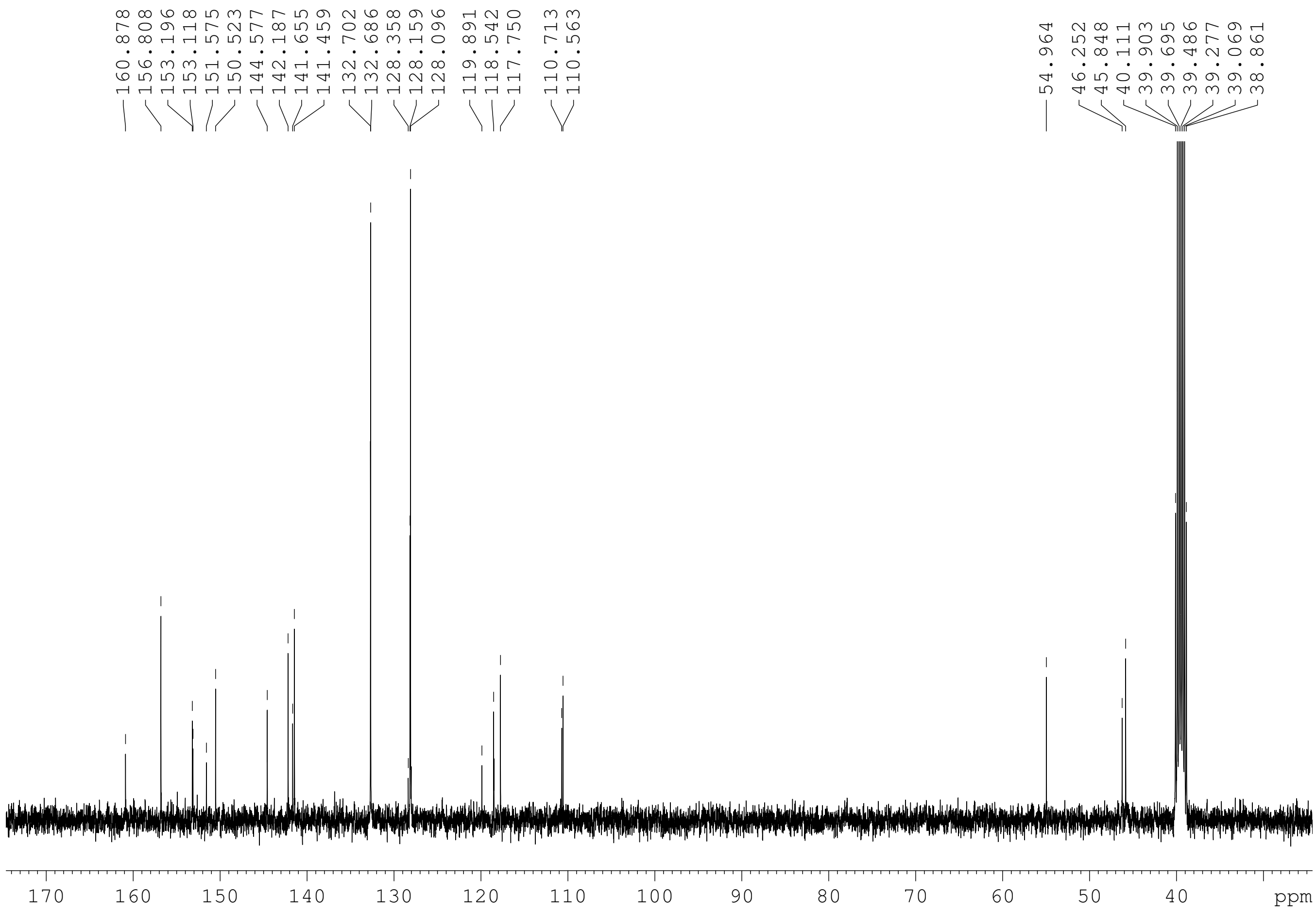

Figure S7. ^{13}C NMR of 4-(6-amino-2-chloropurin-9-ylmethylbenzonitrile (**3**)

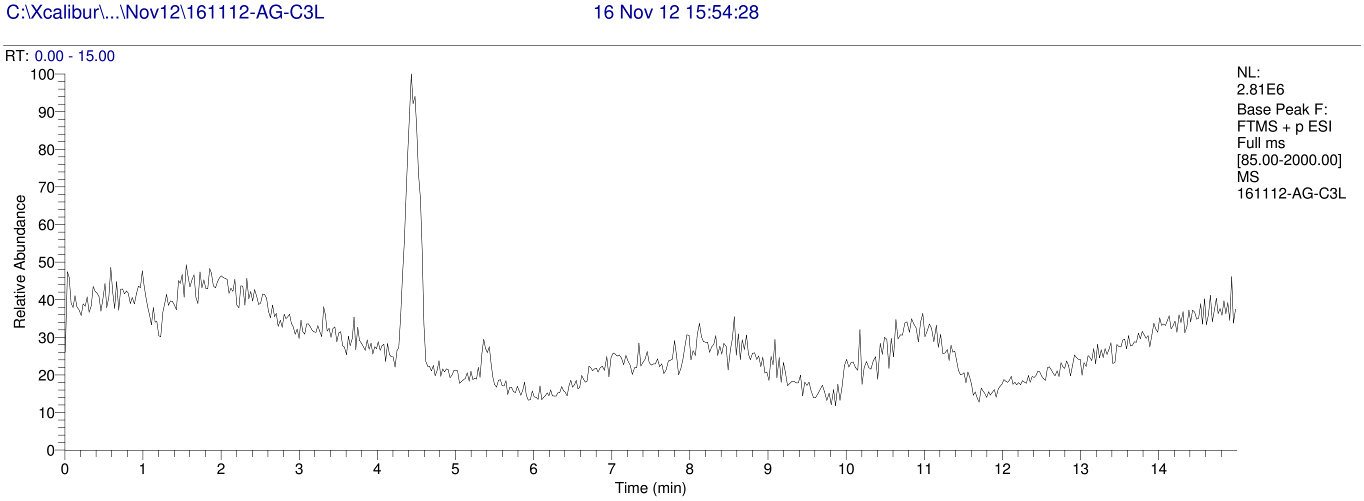

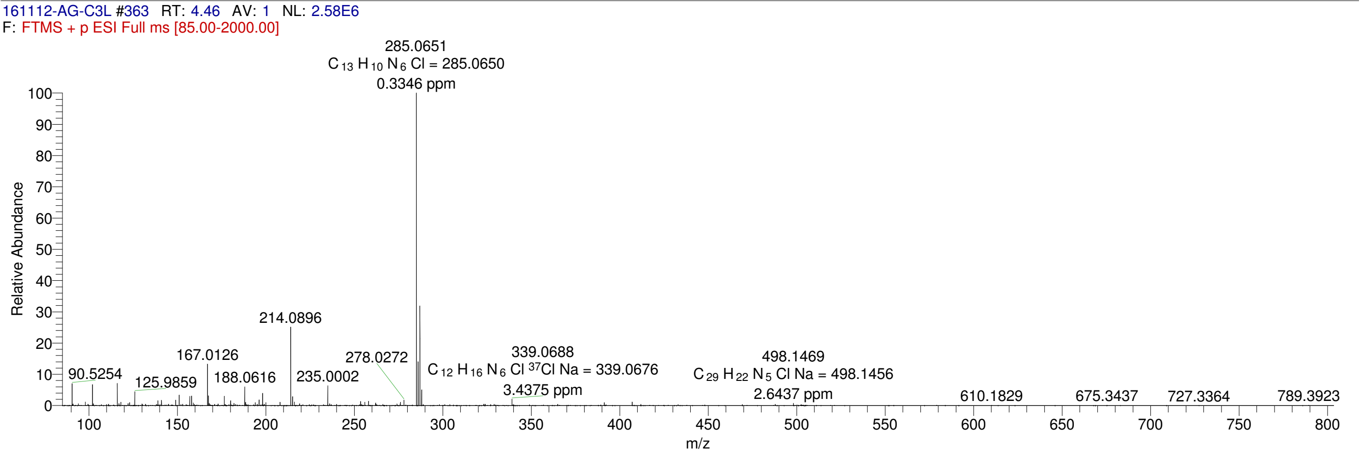

Figure S8. HRMS of 4-(6-amino-2-chloropurin-9-ylmethylbenzonitrile (**3**)

4-[6-amino-2-(2-methoxyethoxy)purin-9-ylmethyl]benzonitrile (**4**)

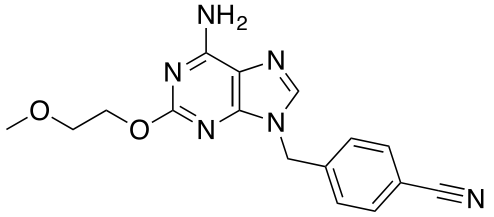

Figure S9. Diagram of 4-[6-amino-2-(2-methoxyethoxy)purin-9-ylmethyl]benzonitrile (**4**)

The sodium salt of 2-methoxy-ethanol was first generated by adding sodium (160 mg, 7 mmol) to 2-methoxy-ethanol (30 mL) with heat. Compound **3** (1g, 3.5 mmol) was dissolved in 2-methoxy-ethanol (300 mL). The mixture was heated to reflux and the sodium 2-methoxy-ethoxide solution added and stirred for 12 h. The mixture was then concentrated to near dryness and the resulting residue was partitioned between EtOAc and H_20. The organic layer was concentrated then subject to flash silica chromatography using MeOH (5%) and DCM. ^{1}H NMR (DMSO-d_6) δ 8.08 (1H, s, NC$\underline{H}$N), 7.83 (2H, d, J = 8.4, 2x *m*-ArH) 7.46 (2H, d, J= 8.4, 2x *o*-ArH), 7.29 (2H, s, NH_2), 5.37 (2H, s, ArC$\underline{H}_2$N), 4.31, (2H, t, J = 4.7, OC$\underline{H}_2$CH$_2$OCH$_3$), 3.59 (2H, t, J = 4.7, OCH$_2$C$\underline{H}_2$OCH$_3$), 3.27 (3H, s, OCH$_2$CH$_2$OC$\underline{H}_3$). ^{13}C NMR (DMSO-d_6) δ161.3, 156.8, 151.1, 142.6, 139.4, 132.6, 128.4, 118.6, 115.1, 110.4, 70.2, 65.3, 58.0, 45.6. HRMS calculated for $C_{16}H_{17}N_6O_2^+$ (MH$^+$) 325.1408 found 325.1408. mp 189-190°c

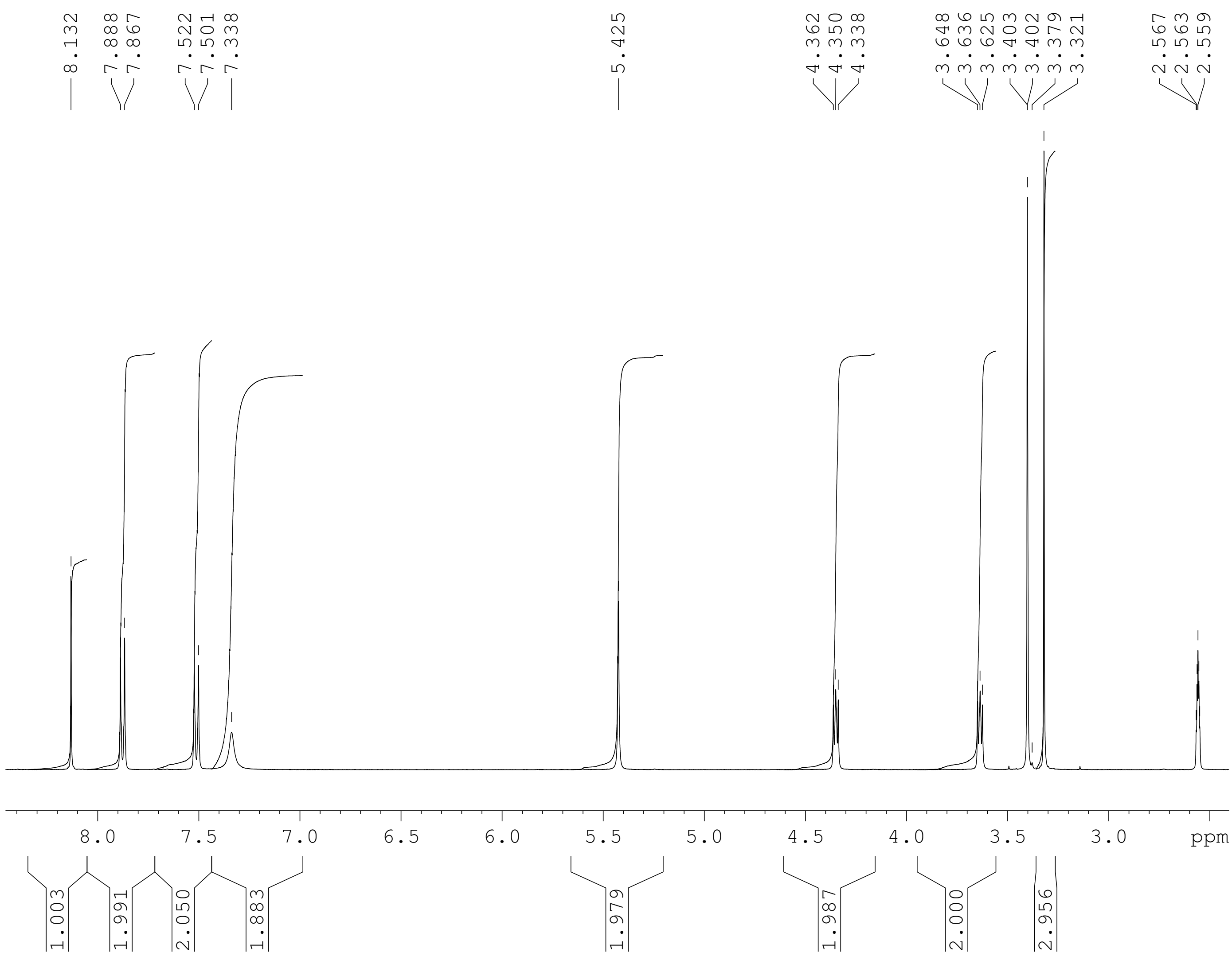

Figure S10. ^{1}H NMR of Diagram of 4-[6-amino-2-(2-methoxyethoxy)purin-9-ylmethyl]benzonitrile (**4**)

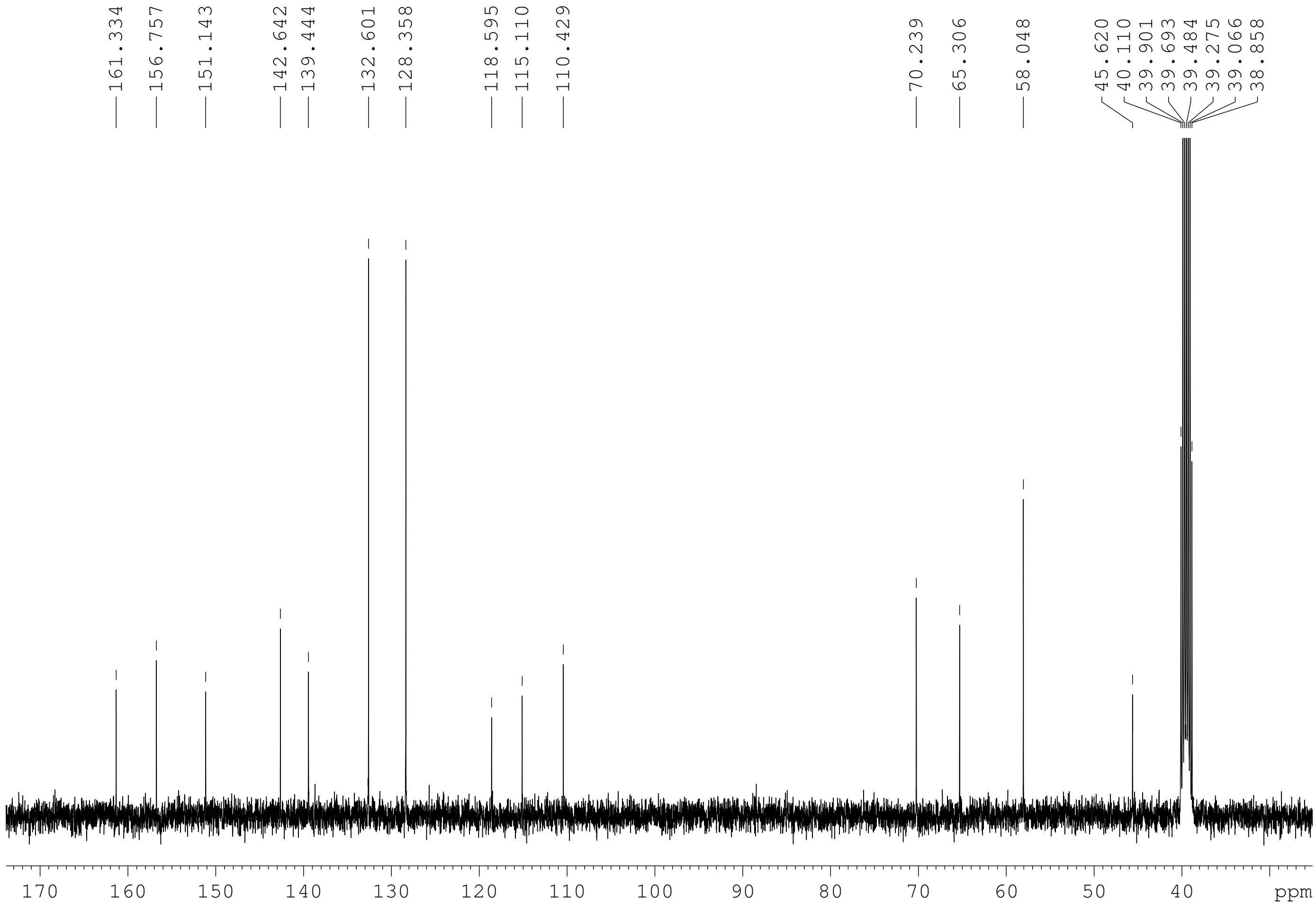

Figure S11. ^{13}C NMR of Diagram of 4-[6-amino-2-(2-methoxyethoxy)purin-9-ylmethyl]benzonitrile (**4**)

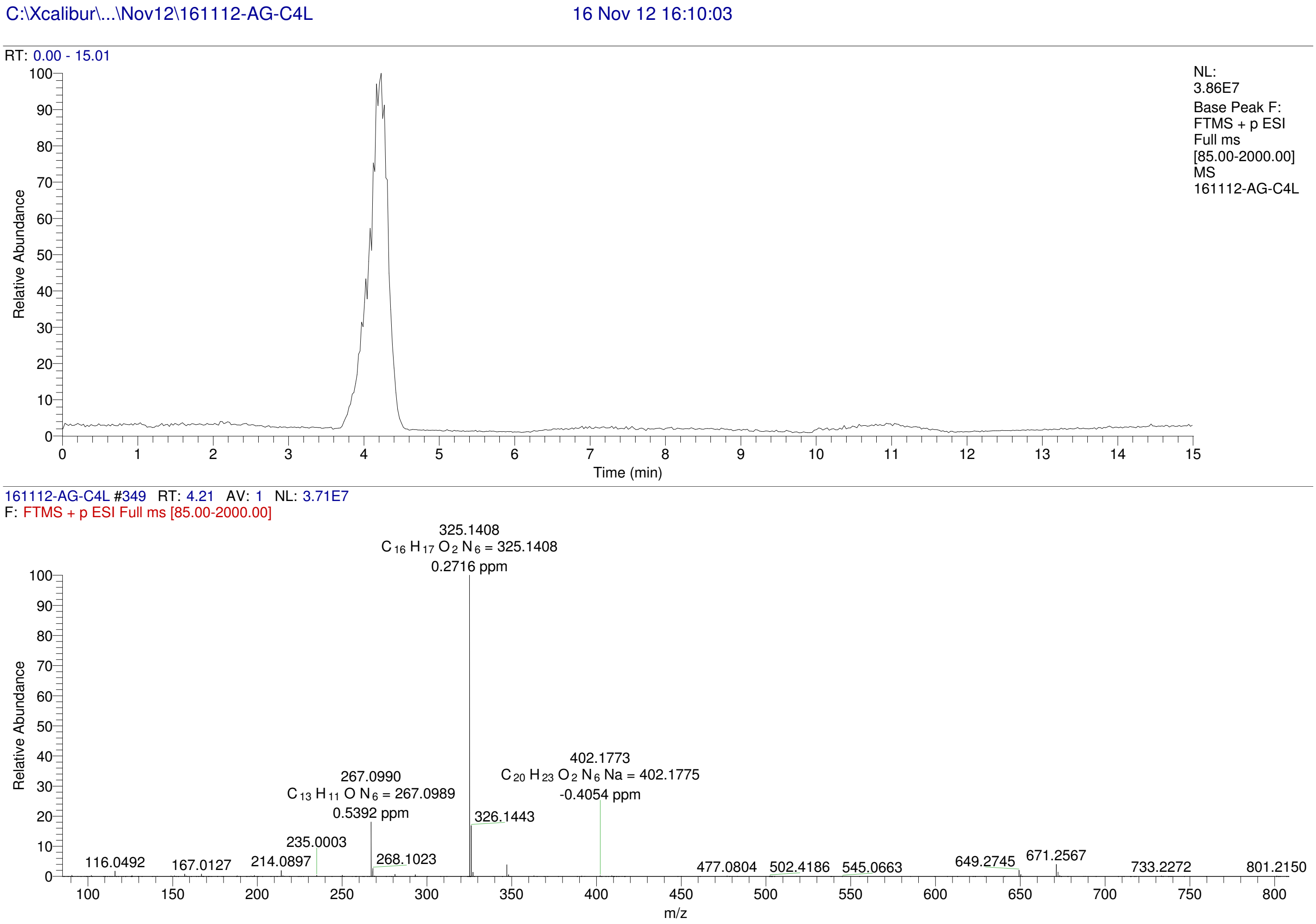

Figure S12. ^{13}C NMR of Diagram of 4-[6-amino-2-(2-methoxyethoxy)purin-9-ylmethyl]benzonitrile (**4**)

4-[6-amino-8-bromo-2-(2-methoxyethoxy)purin-9-ylmethyl]- benzonitrile (**5**)

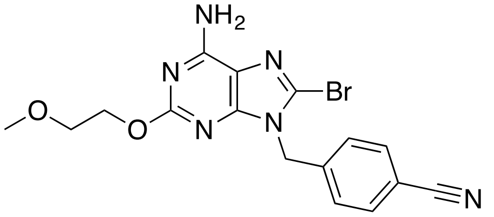

Figure S13. Diagram of 4-[6-amino-8-bromo-2-(2-methoxyethoxy)purin-9-ylmethyl]- benzonitrile (**5**)

Compound **4** (680 mg, 2.1 mmol) was dissolved in DCM (400 mL) and Br_2 (7ml) was added dropwise. The mixture was then stirred overnight at RT. The reaction mixture was extracted using sodium thiosulfate solution (2000 mL, 0.1 M) and then with sodium bicarbonate (500 mL, saturated). The residue from the organic layer was subject to silica gel chromatography using MeOH (3%) in DCM. ^{1}H NMR (DMSO-d_6) δ 7.84 (2H, d, J = 8.4, 2x *m*-ArH), 7.50 (2H, s, NH_2), 7.39 (2H, d, J= 8.4, 2x *o*-ArH), 5.36 (2H, s, Ar$C\underline{H}_2$N), 4.31, (2H, t, J = 4.7, O$C\underline{H}_2CH_2OCH_3$), 3.59 (2H, t, J = 4.7, $OCH_2C\underline{H}_2OCH_3$), 3.27 (3H, s, $OCH_2CH_2OC\underline{H}_3$). ^{13}C NMR (DMSO-$d_6$) δ161.4, 155.7, 152.4, 141.5, 132.7, 127.9, 123.7, 118.5, 110.6, 70.2, 65.5, 58.1, 54.9, 45.9. HRMS calculated for $C_{16}H_{16}BrN_6O_2^+$ (MH^+) 403.0513 found 403.0515. mp 204-205°c

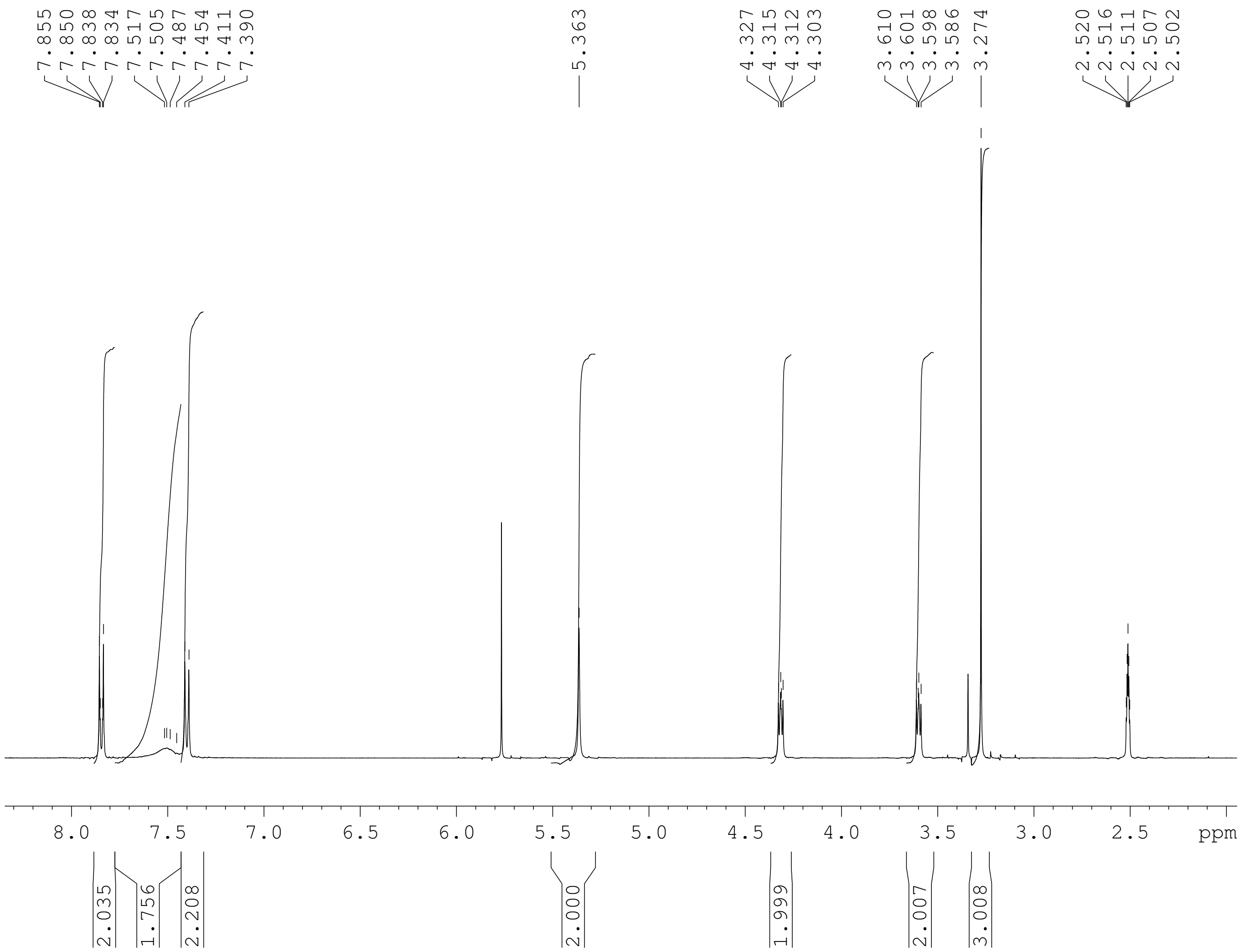

Figure S14. ^{1}H NMR of Diagram of 4-[6-amino-8-bromo-2-(2-methoxyethoxy)purin-9-ylmethyl]- benzonitrile (**5**)

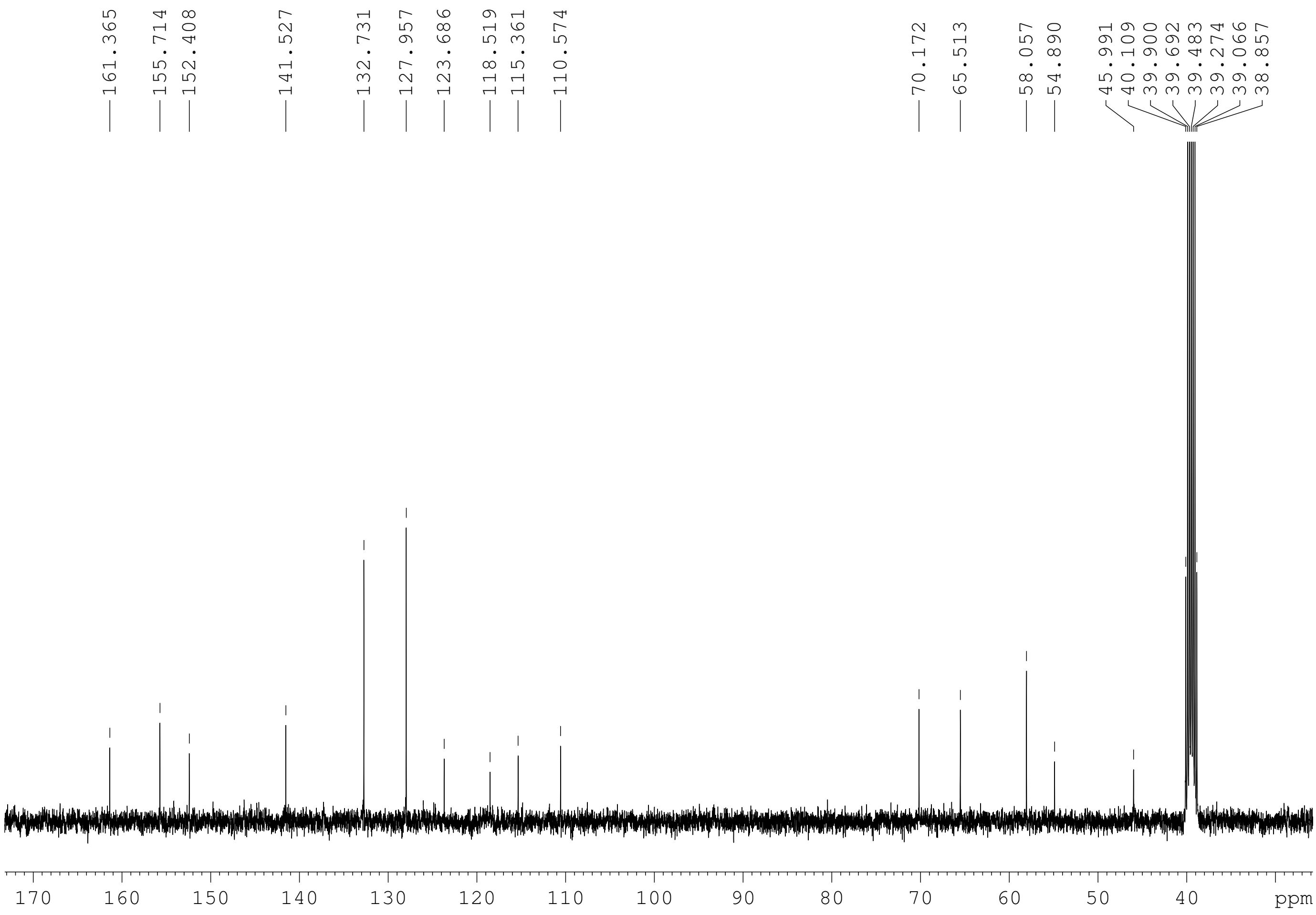

Figure S15. ^{13}C NMR of Diagram of 4-[6-amino-8-bromo-2-(2-methoxyethoxy)purin-9-ylmethyl]- benzonitrile (**5**)

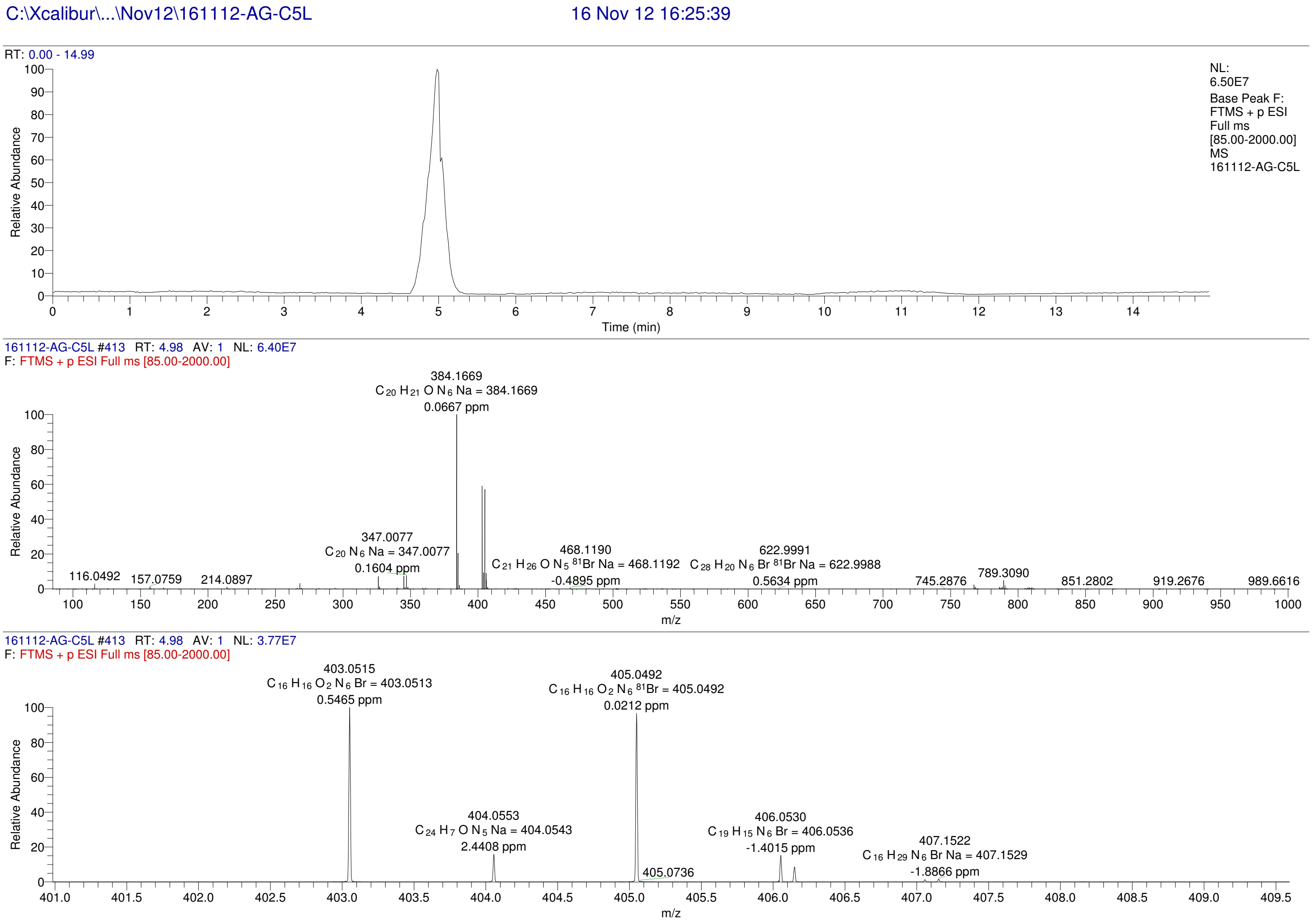

Figure S15. HRMS of Diagram of 4-[6-amino-8-bromo-2-(2-methoxyethoxy)purin-9-ylmethyl]- benzonitrile (**5**)

4-[6-amino-8-methoxy-2-(2-methoxyethoxy)purin-9- ylmethyl]benzonitrile (**6**)

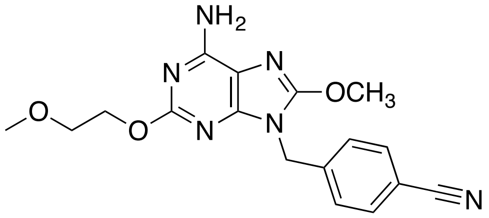

Figure S16. Diagram of 4-[6-amino-8-methoxy-2-(2-methoxyethoxy)purin-9-ylmethyl]benzonitrile (**6**)

Sodium methoxide solution was made by reacting sodium (160 mg, 7 mmol) with dry MeOH (30 mL). Compound **5** (683 mg, 1.7 mmol) was dissolved in dry MeOH (200 mL) by heating, once near reflux the sodium methoxide solution was added and heated to reflux overnight. The mixture was then concentrated *in vacuo* and the residue was subjected to silica gel chromatography using MeOH (5%) in DCM (50%) and EtOAc (45%). ^{1}H NMR (DMSO-d_6) δ 7.82 (2H, d, J = 8.4, 2x *m*-ArH), 7.40 (2H, d, J= 8.4, 2x *o*-ArH), 6.93 (2H, s, NH_2), 5.14 (2H, s, ArC$\underline{H}_2$N), 4.27, (2H, t, J = 4.7, OC$\underline{H}_2$$CH_2OCH_3$), 4.04, (3H, s, OC$\underline{H}_3$), 3.58 (2H, t, J = 4.7, OCH_2C$\underline{H}_2$OCH_3), 3.27 (3H, s, O$CH_2$$CH_2OC\underline{H}_3$). ^{13}C NMR (DMSO-$d_6$) δ159.9, 154.5, 153.3, 150.8, 142.2, 132.6, 128.0, 118.6, 110.3, 110.0, 70.3, 65.1, 58.0, 56.9, 43.4 HRMS calculated for $C_{17}H_{19}N_6O_3^+$ (MH^+) 355.1513 found 355.1513
IR (v, cm^{-1}) : 3432 , 3325, 3234, 2928, 2232, 1630, 1600, 1564
mp166-167°c

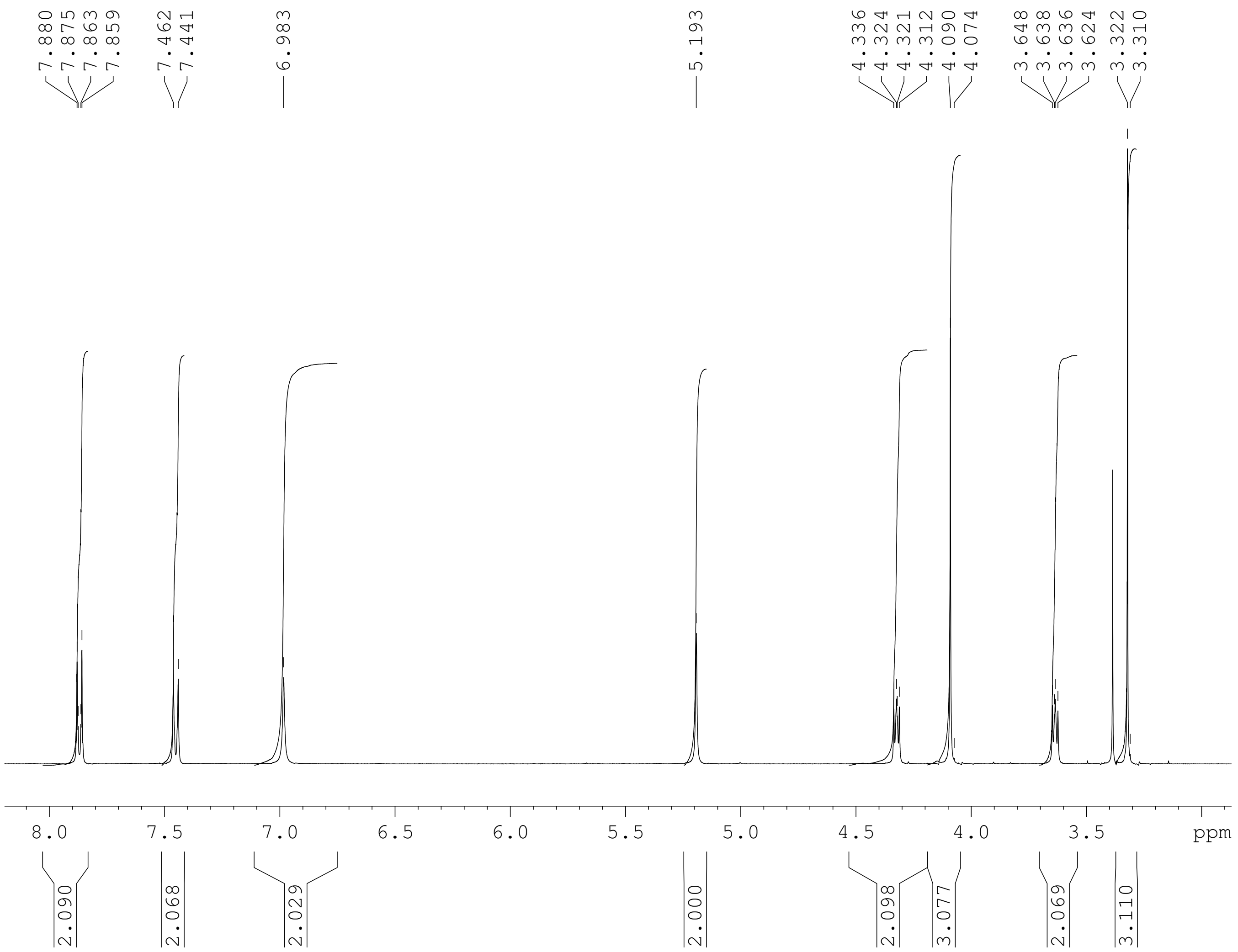

Figure S17. ^{1}H NMR of 4-[6-amino-8-methoxy-2-(2-methoxyethoxy)purin-9-ylmethyl]benzonitrile (**6**)

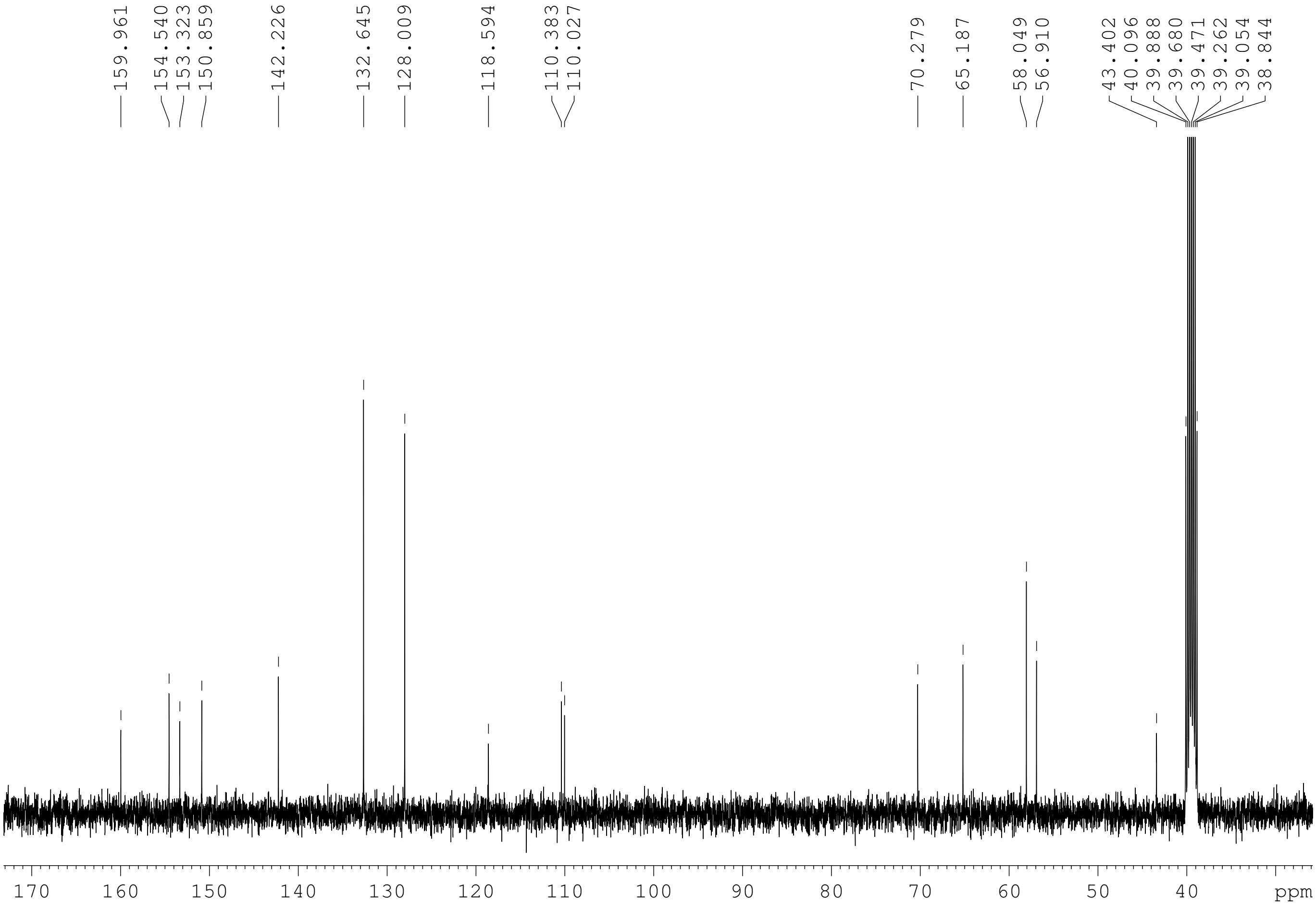

Figure S18. ^{13}C NMR of 4-[6-amino-8-methoxy-2-(2-methoxyethoxy)purin-9-ylmethyl]benzonitrile (**6**)

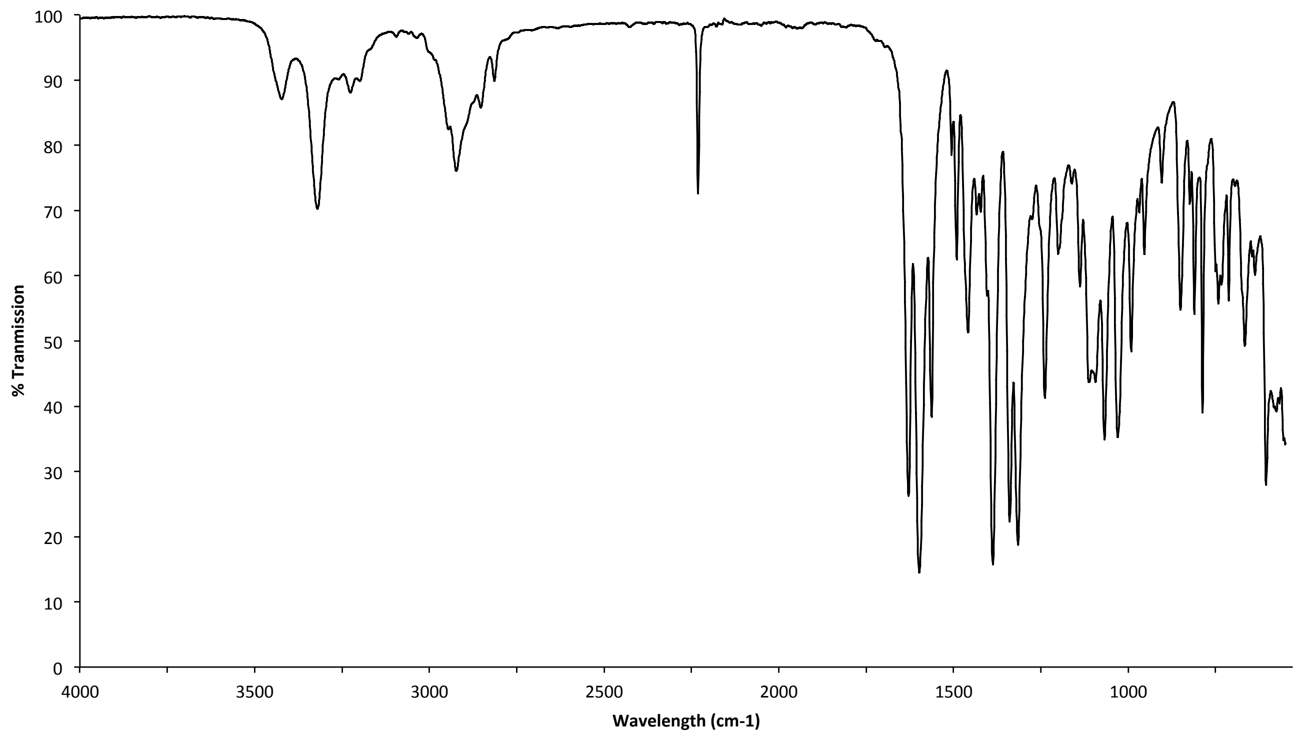

Figure S19. FTIR of 4-[6-amino-8-methoxy-2-(2-methoxyethoxy)purin-9-ylmethyl]benzonitrile (**6**)

C:\Xcalibur\...\Nov12\161112-AG-C6L 16 Nov 12 16:56:53

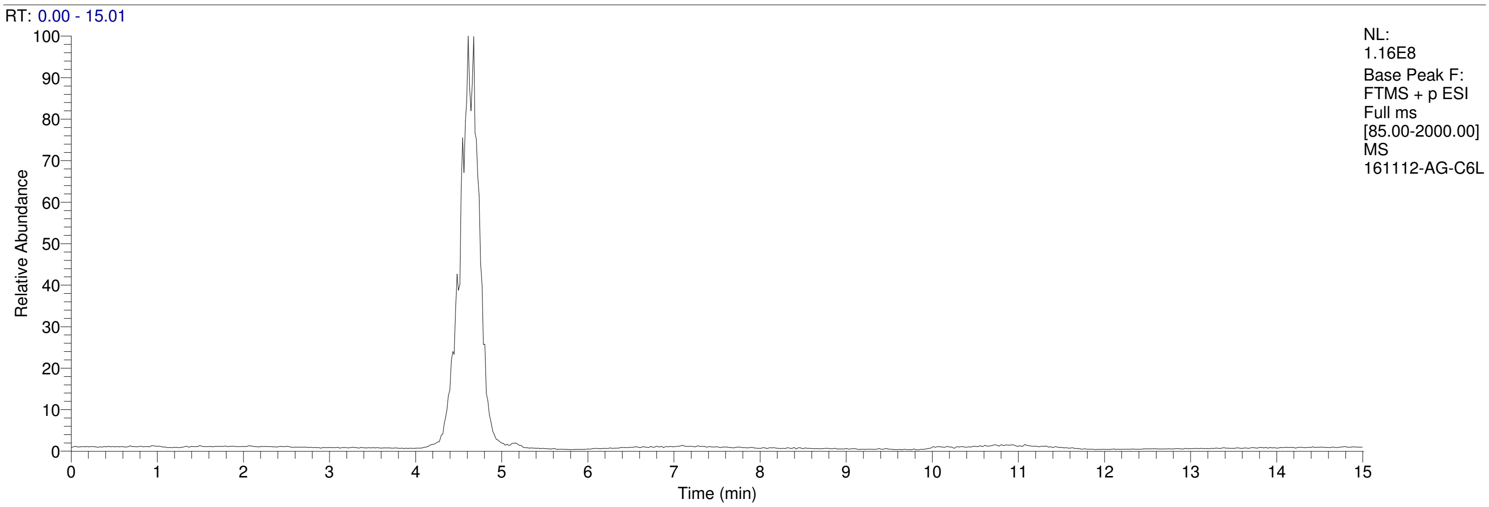

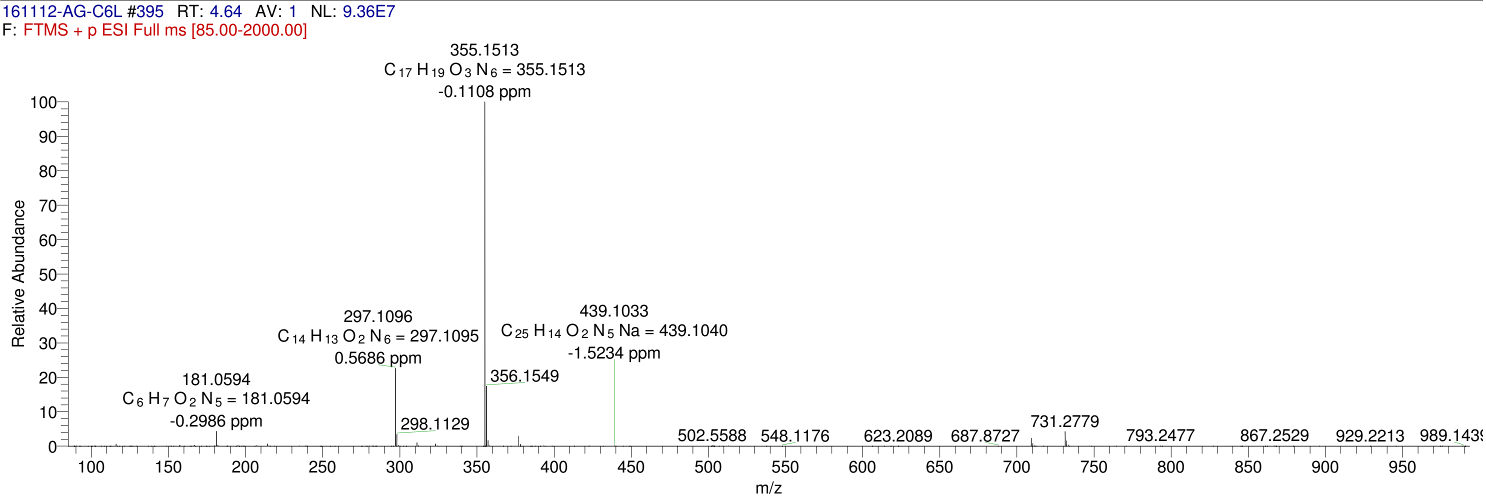

Figure S20. HRMS of 4-[6-amino-8-methoxy-2-(2-methoxyethoxy)purin-9-ylmethyl]benzonitrile (**6**)

Synthesis of 4-{[6-Amino-2-(2-methoxyethoxy)-8-oxo-7*H*-purin-9(8*H*)-yl]methyl}benzoic Acid (**7**)

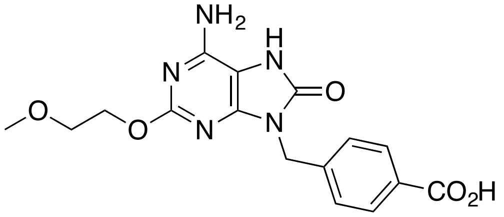

Figure S21. Diagram of 4-{[6-Amino-2-(2-methoxyethoxy)-8-oxo-7*H*-purin-9(8*H*)- yl]methyl}benzoic Acid (7)

Compound **6** (0.3 mmol, 100 mg) was dissolved in EtOH (10ml) and NaOH (10 M, 10 mL). The mixture was heated to reflux for aprox 8 h. Once cool, the mixture was acidified to pH 2 with HCl and extracted with DCM (3x20 mL) dried with MgSO4 and concentrated. This mixture was then dissolved in MeCN (25 mL) with NaI (0.14 g, 0.96 mmol). To this solution TMS-Cl (12 µL, 0.96 mmol) was added dropwise with stirring and heated to 40 C for 4 H. Once cooled the product was filtered washed with H2O (20 mL) and then Et2O (20 mL).

^{1}H NMR (DMSO-d_6) δ 12.94 (1H, s, ArC(O)O<u>H</u>), 10.03 (1H, s, NHC(O)N), 7.89 (2H, d *J* = 8.2, 2x *o*-ArH), 7.39 (2H, d, *J* = 8.2, 2x *m*-ArH), 6.51 (2H, s, NH_2), 4.94 (2H, s, ArC<u>H$_2$</u>N), 4.25 (2H, t, *J* = 4.2, OC<u>H$_2$</u>CH$_2$OCH$_3$), 3.57 (2H, t, *J* = 4.2, OCH$_2$C<u>H$_2$</u>OCH$_3$), 3.26 (3H, s, OCH$_2$CH$_2$OC<u>H$_3$</u>). ^{13}C NMR (DMSO-d_6) δ167.0, 159.7, 152.2, 149.1, 147.8, 142.0, 129.6, 127.4, 98.3, 70.2, 65.3, 58.0, 42.12. HRMS calculated for C-$_{16}H_{18}O_5N_5^+$ (MH^+) 360.1302 found 360.1302.

IR (v, cm^{-1}) : 3351, 3165, 2956 (Broad), 1703, 1630, 1603

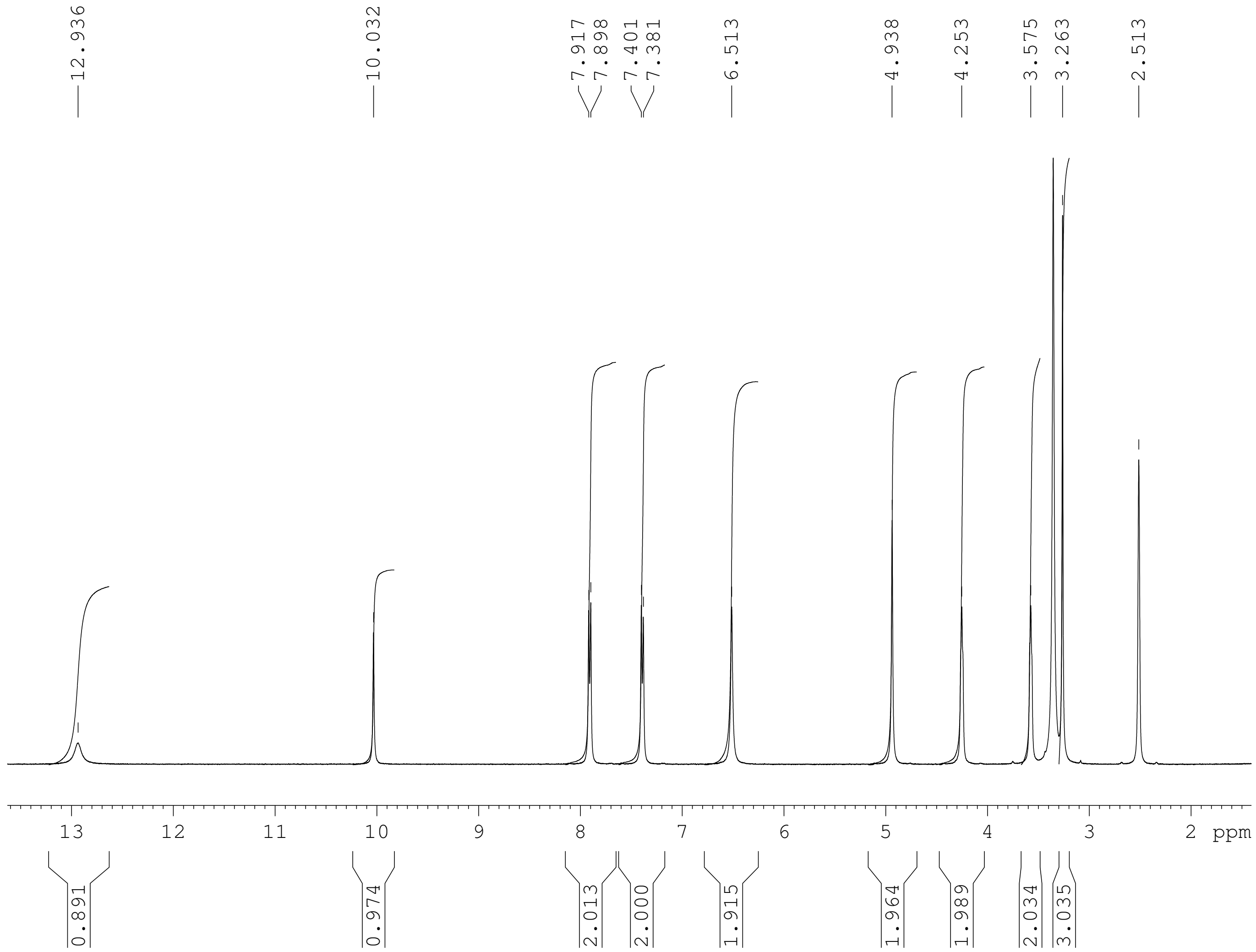

Figure S22. ^{1}H NMR of 4-{[6-Amino-2-(2-methoxyethoxy)-8-oxo-7*H*-purin-9(8*H*)- yl]methyl}benzoic Acid (**7**)

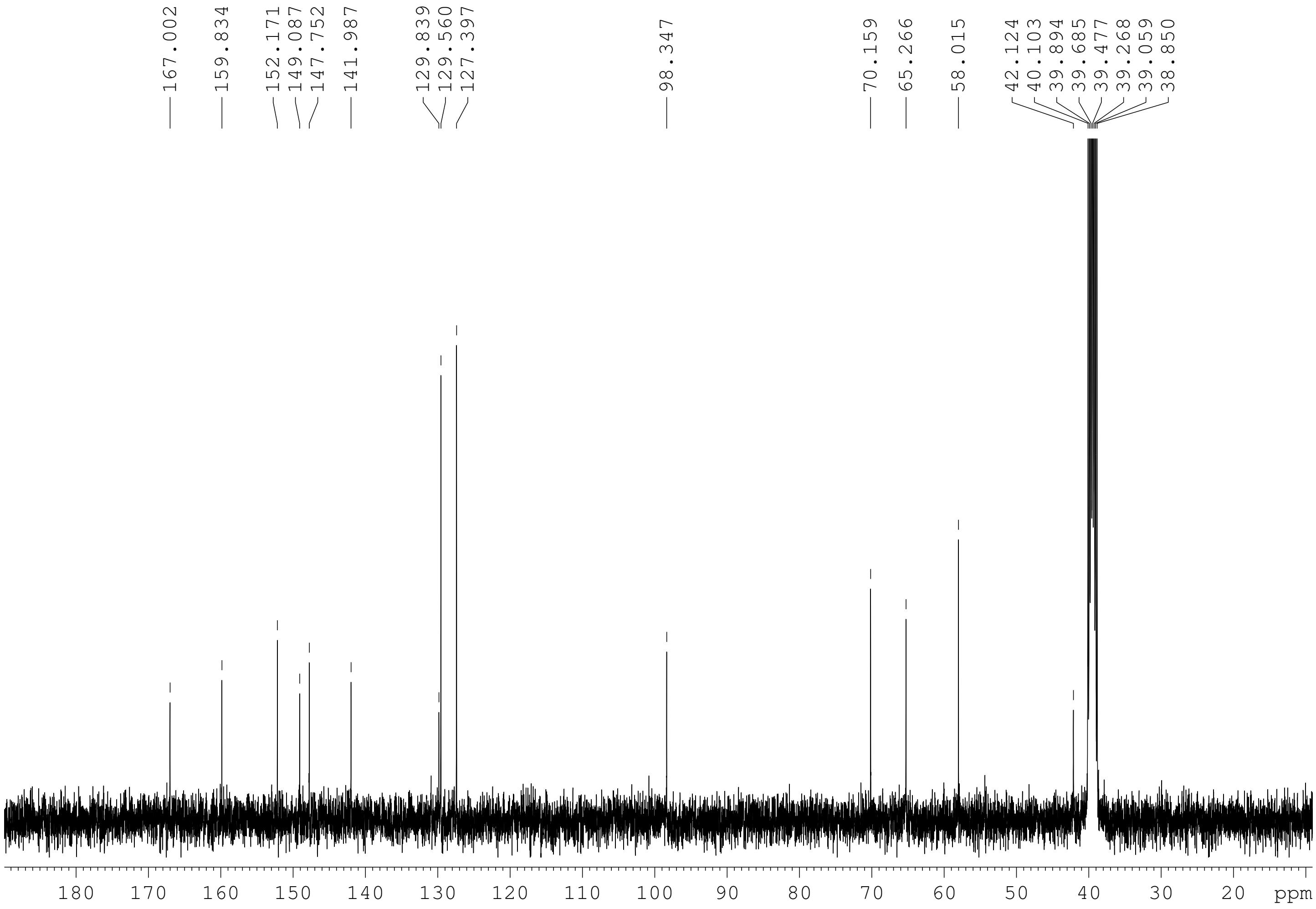

Figure S23. ^{13}C NMR of 4-{[6-Amino-2-(2-methoxyethoxy)-8-oxo-7*H*-purin-9(8*H*)- yl]methyl}benzoic Acid (**7**)

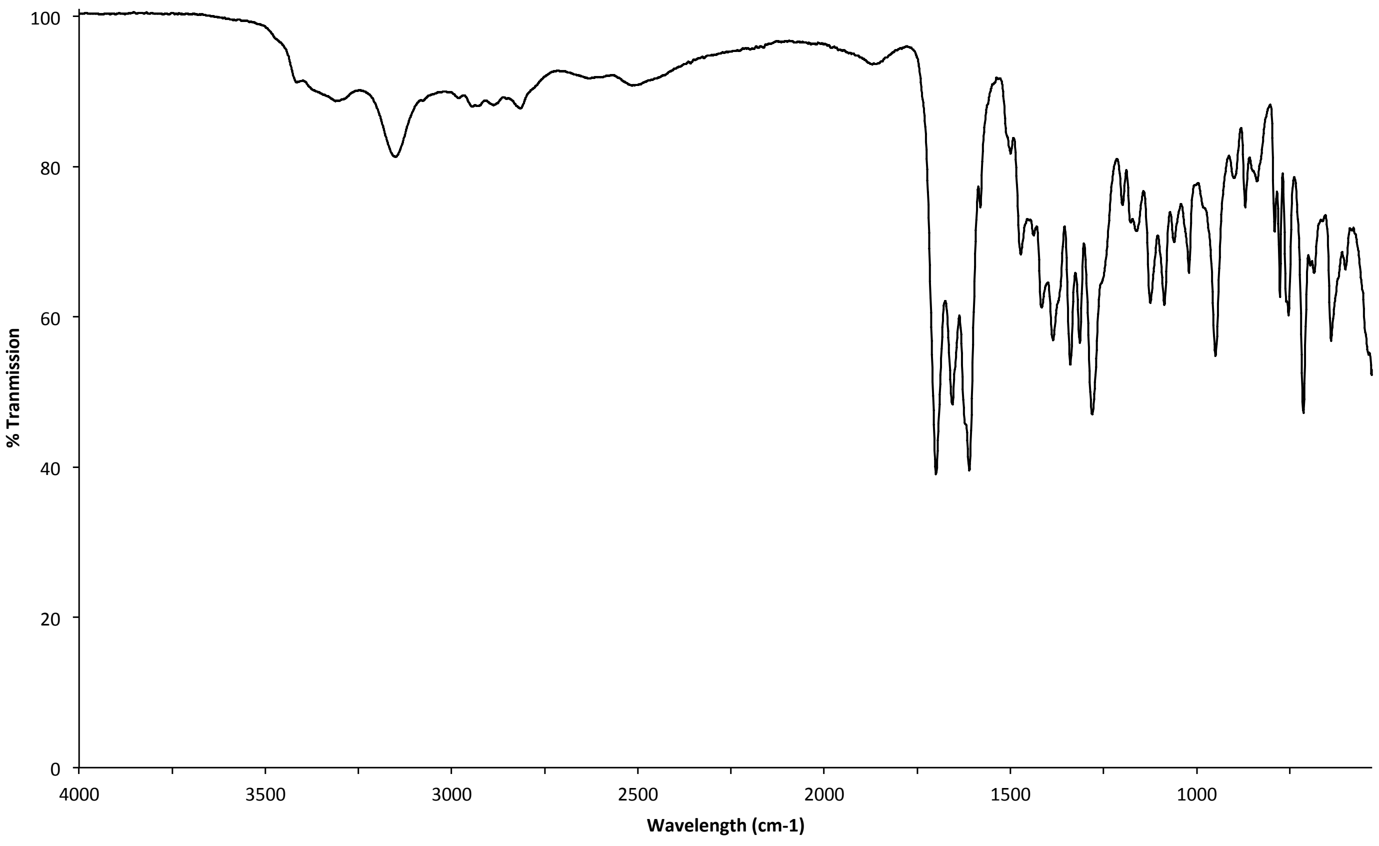

Figure S24. FTIR of 4-{[6-Amino-2-(2-methoxyethoxy)-8-oxo-7*H*-purin-9(8*H*)-yl]methyl}benzoic Acid (**7**)

C:\Xcalibur\...\Feb13\070213-AG-C3M 08 Feb 13 11:36:06

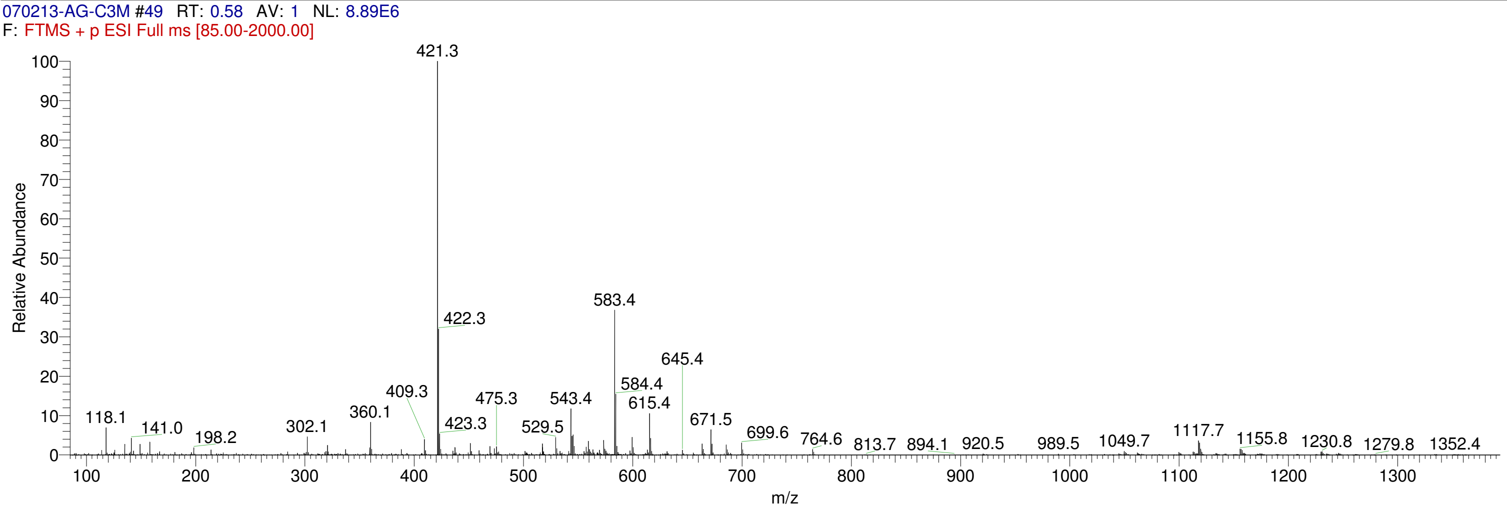

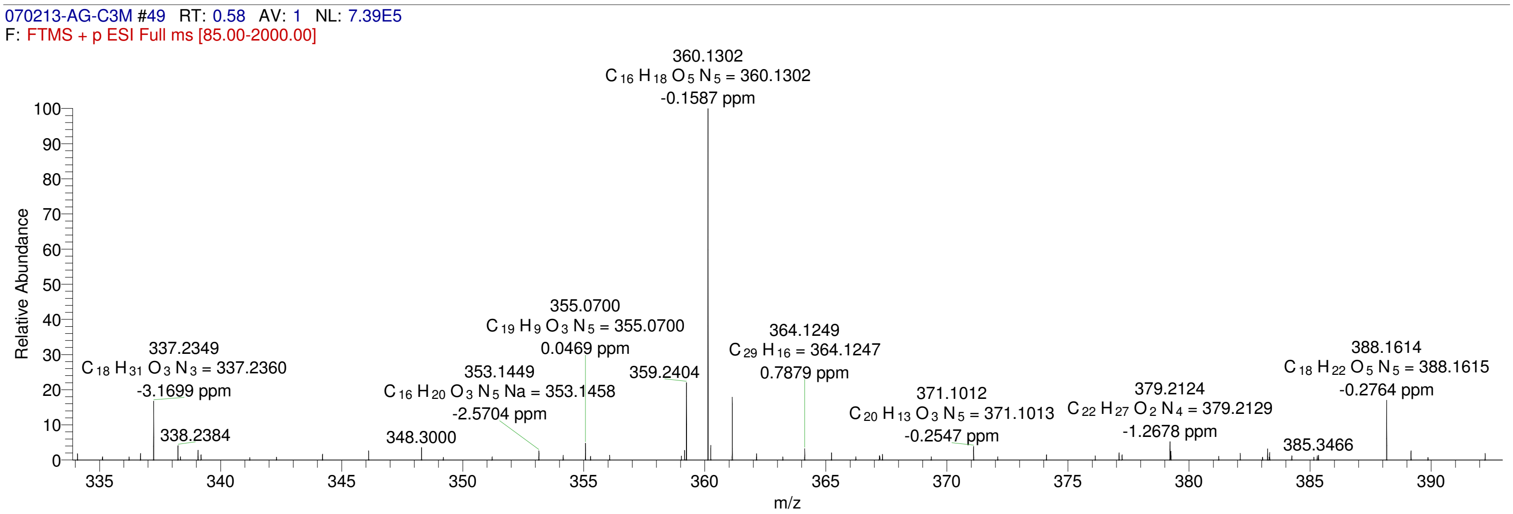

Figure S24. HRMS of 4-{[6-Amino-2-(2-methoxyethoxy)-8-oxo-7*H*-purin-9(8*H*)- yl]methyl}benzoic Acid (**7**)

Synthesis of (2R)-3-(((2-(4-((6-amino-2-(2-methoxyethoxy)-8-oxo-7,8-dihydro-9H-purin-9-yl)methyl)benzamido)ethoxy)(hydroxy)phosphoryl)oxy)propane-1,2-diyl dioleate (**8**)

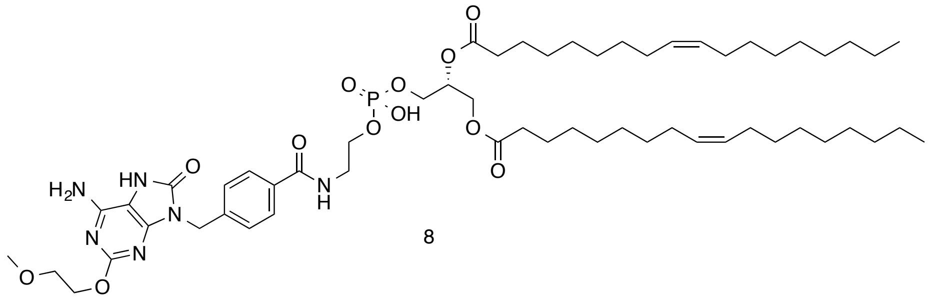

8

Figure S25. Diagram of (2R)-3-(((2-(4-((6-amino-2-(2-methoxyethoxy)-8-oxo-7,8-dihydro-9H-purin-9-yl)methyl)benzamido)ethoxy)(hydroxy)phosphoryl)oxy)propane-1,2-diyl dioleate (**8**)

To a solution of compound **7** (0.022 g, 0.06 mmol) in anhydrous DMF (1 mL) was added *O*-(7-azabenzotriazol-1-yl)-*N*,*N*,*N′*,*N′*-tetramethyluronium hexafluorophosphate (HATU) (0.026 g, 0.067 mmol) and anhydrous triethylamine (TEA) (17.0 µL, 0.12 mmol).
A solution of 1,2- dioleoyl-*sn*-glycero-3-phosphoethanolamine (DOPE) (0.05 g, 0.067 mmol) in anhydrous 1:1 DCM/DMF (1 mL) was prepared and slowly added to the reaction mixture. The reaction mixture was stirred at room temperature until completion and then evaporated in vacuo. The product was purified by flash chromatography using 15% MeOH in DCM.
^{1}H NMR (DMSO-d_6, 700 MHz, 45 °C): δ 10.35 (1H, s, N$\underline{H}$C(O)N), 9.97 (1H, bs, ArC(O)N$\underline{H}CH_2CH_2$), 9.37 (1H, bs, $PO_3O\underline{H}$), 7.86 (2H, d, *J* 8.2, 2x *o*-Ar$\underline{H}$), 7.34 (2H, d, *J* 8.2, 2x *m*-Ar$\underline{H}$), 6.60 (2H, bs, NH_2), 5.31 (4H, m, 2x $CH_2C\underline{H}C\underline{H}CH_2$), 5.09 (1H, m, $POCH_2C\underline{H}CH_2O$), 4.89 (2H, s, $NC\underline{H}_2Ar$), 4.29 (1H, dd, *J* 3.1, 12.0, $C(O)NHC\underline{H}_2CH_2OPO_3$) 4.25 (2H, m, $OC\underline{H}_2CH_2OCH_3$), 4.09 (1H, m, $C(O)NHC\underline{H}_2CH_2OPO_3$), 3.84 (2H, bs, $POC\underline{H}_2CHCH_2O$), 3.81 (2H, bs, $POCH_2CHC\underline{H}_2O$) 3.57 (2H, m, $OCH_2C\underline{H}_2OCH_3$), 3.26 (3H, s, $OCH_2CH_2OC\underline{H}_3$), 3.17, (1H, d, J 5.1), 2.24 (4H, m, 2x $OC(O)C\underline{H}_2CH_2$), 1.95 (8H, m, 2x $CH_2C\underline{H}_2CHCHC\underline{H}_2CH_2$), 1.48 (4H, m, 2x $OC(O)CH_2C\underline{H}_2$), 1.23 (40H, m, Lipid tail $C\underline{H}_2$), 0.85 (6H, m, 2x $CH_2CH_2C\underline{H}_3$).
^{13}C NMR (DMSO-d_6, 175 MHz) δ 173.1, 172.8, 160.4, 152.7, 149.6, 148.5, 140.7, 134.0, 130.2, 128.0, 127.8, 99.0, 70.8, 62.9, 58.7, 46.0, 42.7, 40.6, 34.2, 34.0, 31.9, 29.70, 29.69, 29.4, 29.3, 29.23, 29.19, 29.12, 29.10, 29.03, 29.0, 19.2, 14.5.
HRMS calculated for $C_{57}H_{94}N_6O_{12}P^+$ (MH^+) 1085.6662 found 1085.6677

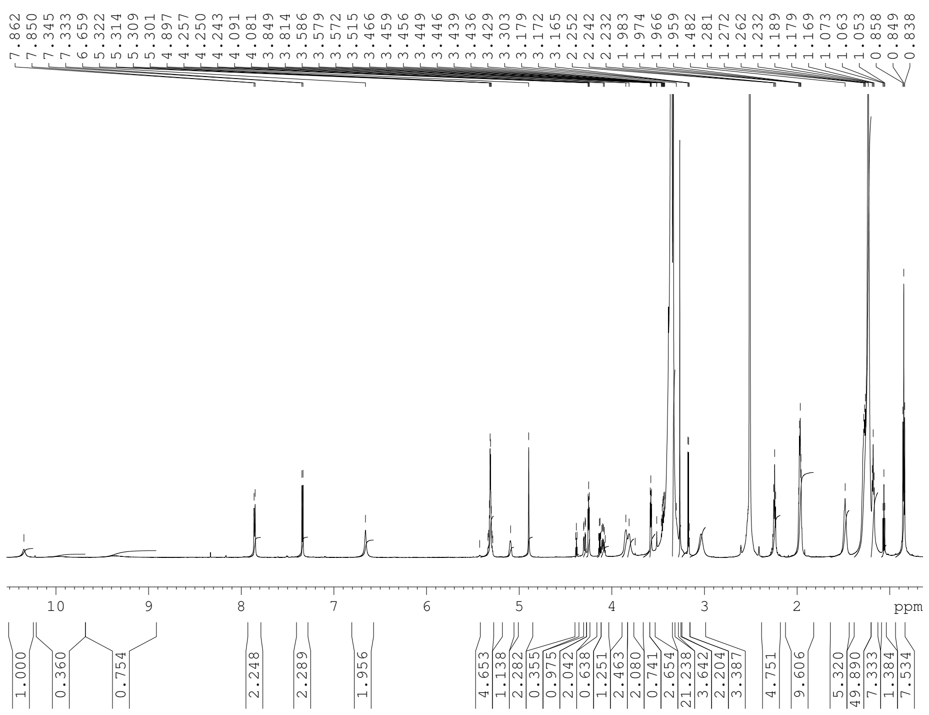

Figure S26. ^{1}H NMR of (2R)-3-(((2-(4-((6-amino-2-(2-methoxyethoxy)-8-oxo-7,8-dihydro-9H-purin-9-yl)methyl)benzamido)ethoxy)(hydroxy)phosphoryl)oxy)propane-1,2-diyl dioleate (**8**)

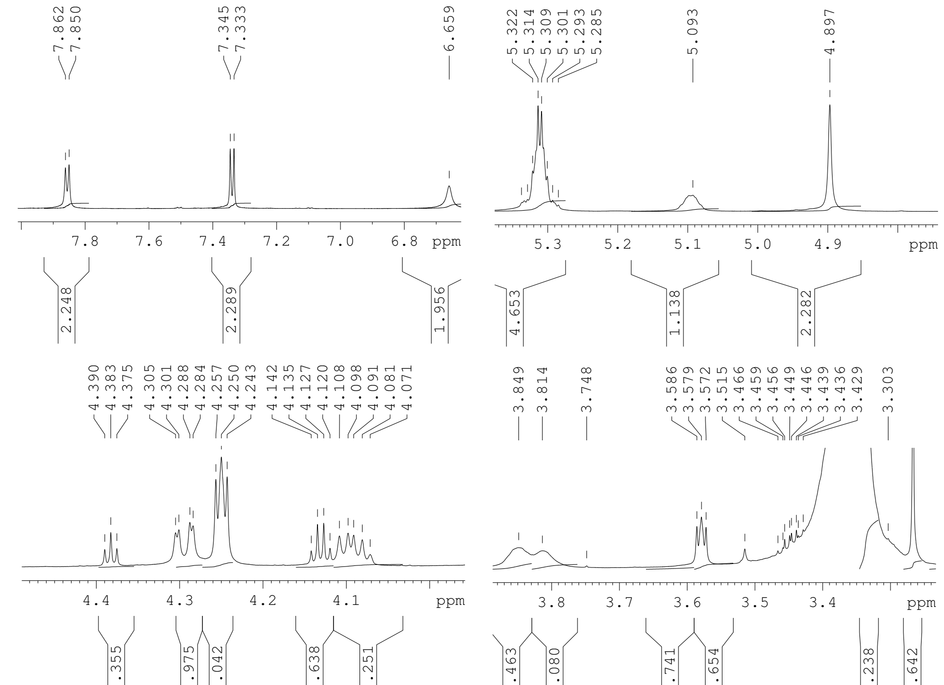

Figure S27. Zooms from ^{1}H NMR of (2R)-3-(((2-(4-((6-amino-2-(2-methoxyethoxy)-8-oxo-7,8-dihydro-9H-purin-9-yl)methyl)benzamido)ethoxy)(hydroxy)phosphoryl)oxy)propane-1,2-diyl dioleate (**8**)

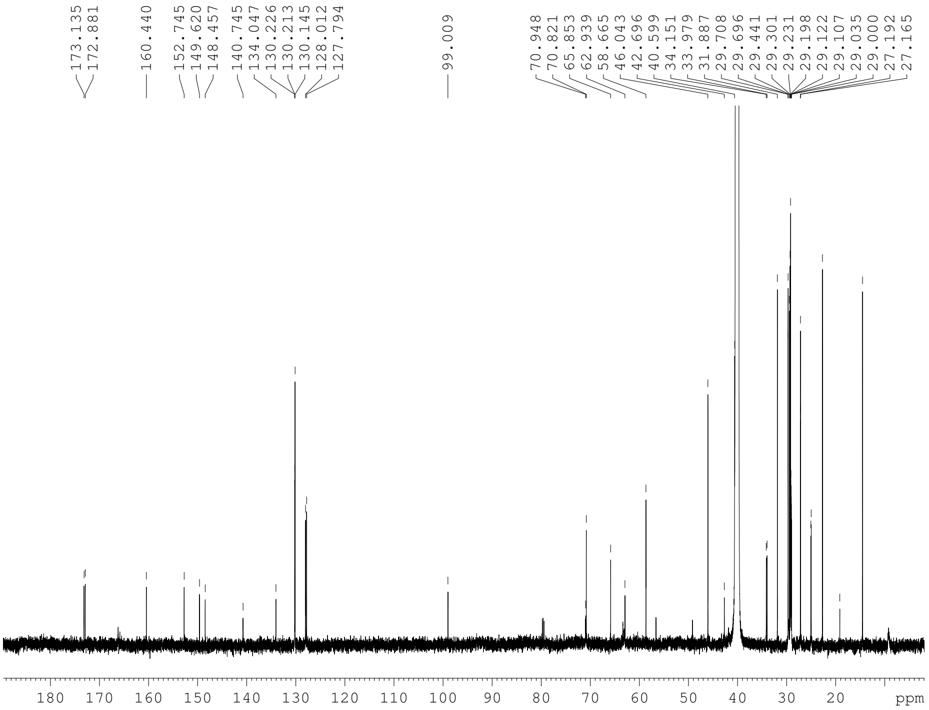

Figure S28. ^{13}C NMR of (2R)-3-(((2-(4-((6-amino-2-(2-methoxyethoxy)-8-oxo-7,8-dihydro-9H-purin-9-yl)methyl)benzamido)ethoxy)(hydroxy)phosphoryl)oxy)propane-1,2-diyl dioleate (**8**)

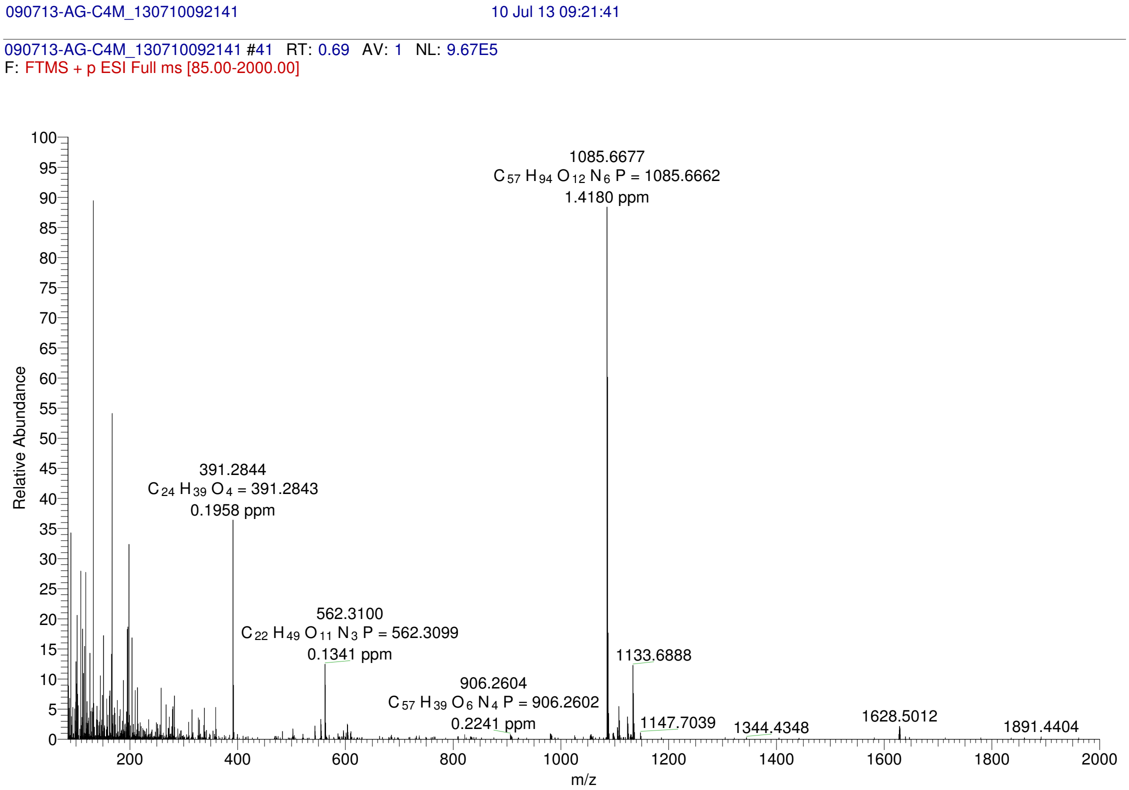

Figure S29. HRMS of (2R)-3-(((2-(4-((6-amino-2-(2-methoxyethoxy)-8-oxo-7,8-dihydro-9H-purin-9-yl)methyl)benzamido)ethoxy)(hydroxy)phosphoryl)oxy)propane-1,2-diyl dioleate (**8**)

FTIR of TLR 7-DOPE conjugate

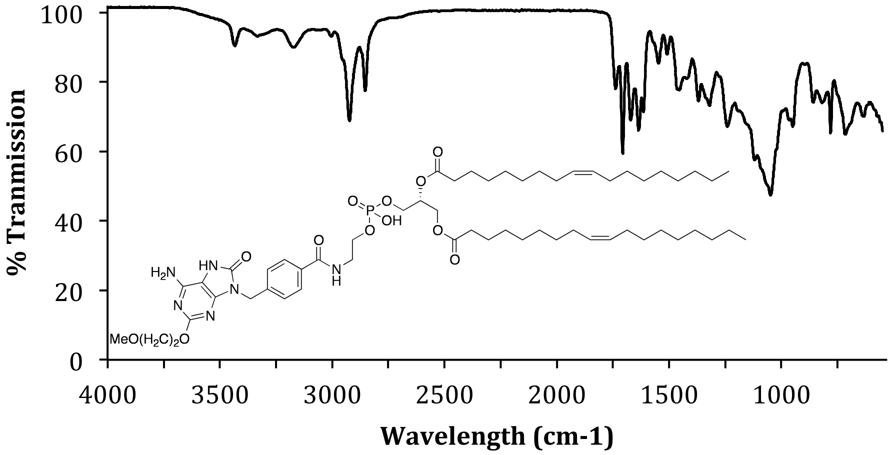

FTIR of unconjugated DOPE

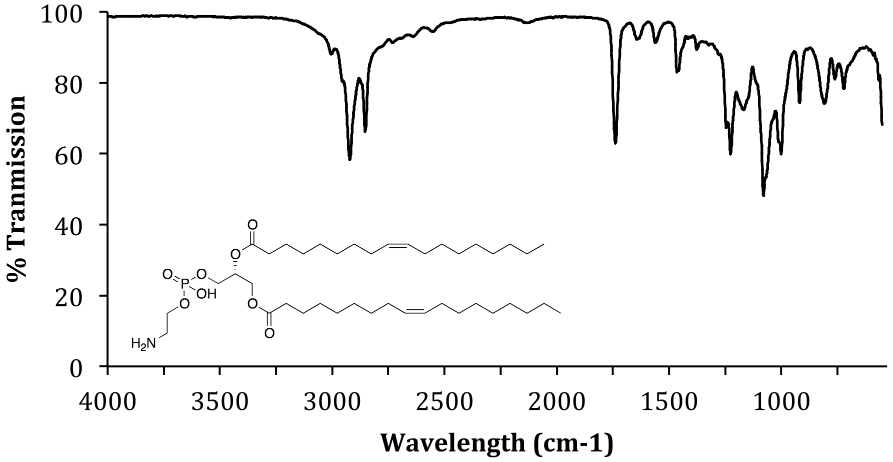

IR spectra of unconjugated TLR 7 agonist

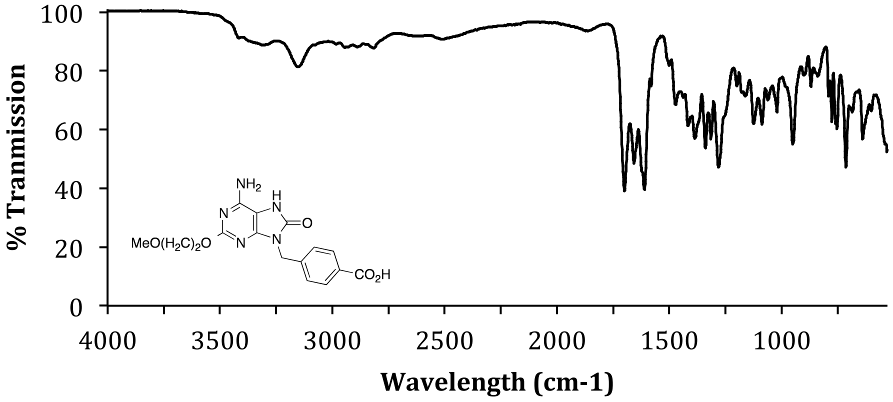

Figure S30. FTIR of TLR7a-DOPE conjugae (2R)-3-(((2-(4-((6-amino-2-(2-methoxyethoxy)-8-oxo-7,8-dihydro-9H-purin-9-yl)methyl)benzamido)ethoxy)(hydroxy)phosphoryl)oxy)propane-1,2-diyl dioleate (**8**) and constituent unconjugated components

Synthesis of 6-amino-9-benzyl-2-(2-methoxyethoxy)-7*H*-purin-8(9*H*)-one (**9**)

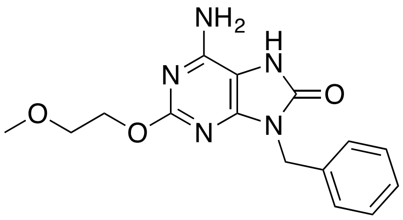

Figure S31. Diagram of Synthesis of 6-amino-9-benzyl-2-(2-methoxyethoxy)-7*H*-purin-8(9*H*)-one (**9**)

6-amino-9-benzyl-2-(2-methoxyethoxy)-7*H*-purin-8(9*H*)-one was synthesised according to the protocol [1] and obtained as white powder.
^{1}H NMR (DMSO-d_6) δ 9.95 (1H, s, NHC(O)N), 7.31 (5H, m, ArH), 6.58 (2H, s, NH_2), 4.86 (2H, s, $ArCH_2N$), 4.26 (2H, t, J = 4.7, $OCH_2CH_2OCH_3$), 3.58 (2H, t, J = 4.7, $OCH_2CH_2OCH_3$), 3.27 (3H, s, $OCH_2CH_2OCH_3$).
^{13}C NMR (DMSO-*d*6) δ159.8, 152.1, 149.1, 147.7, 137.1, 128.4, 127.4, 127.3, 98.3, 70.2, 65.2, 58.0, 42.3. HRMS calculated for $C_{15}H_{18}N_5O_3^+$ (MH^+) 316.1404 found 316.1405 mp decomposition.

Single crystal structure of unmodified TLR7 agonist
A single crystal of the unmodified TLR7 agonist (**9**) was obtained by slow evaporation from DMSO. The crystal data were collected at 151K with copper radiation using an Oxford Diffraction Gemini diffractometer. Full details of the crystal structure are contained in the CIF which has been deposited (deposition number 1825910) with the Cambridge Crystallographic Data Centre *.

* These data can be obtained free of charge via www.ccdc.cam.ac.uk/data_request/cif, or by emailing data_request@ccdc.cam.ac.uk, or by contacting The Cambridge Crystallographic Data Centre, 12, Union Road, Cambridge CB2 1EZ, UK

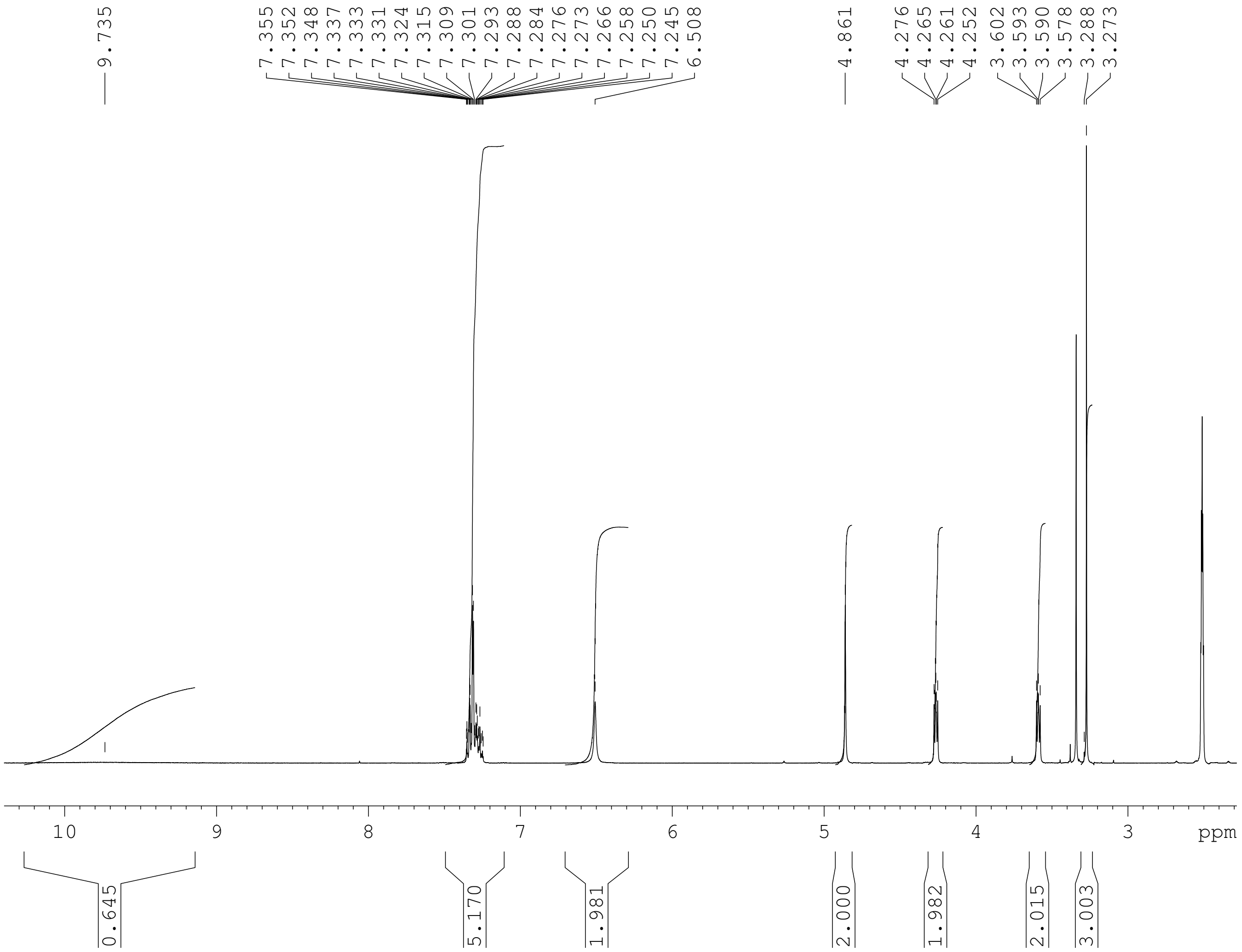

Figure S32. ^{1}H NMR spectra of 6-amino-9-benzyl-2-(2-methoxyethoxy)-7*H*-purin-8(9*H*)-one (**9**)

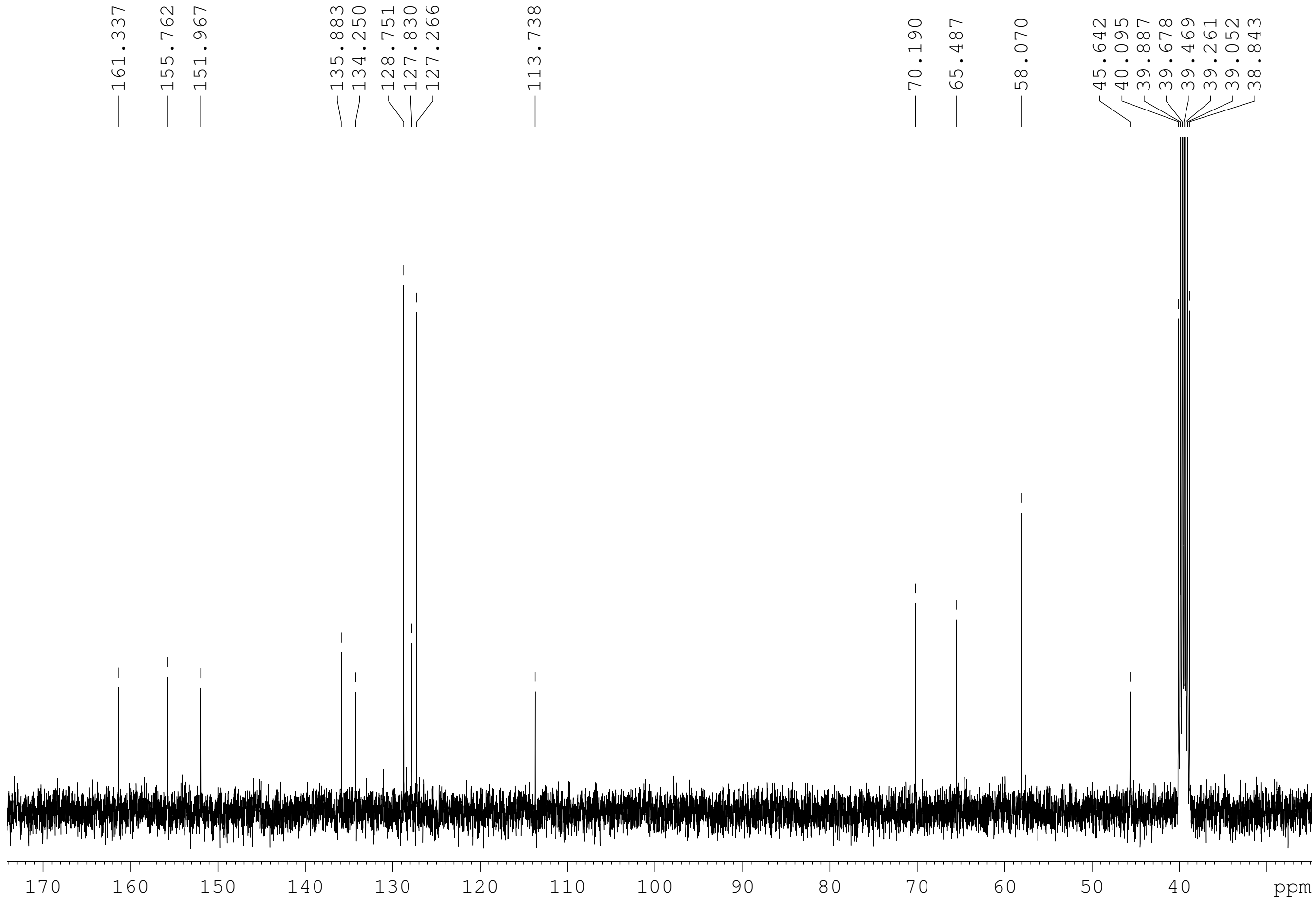

Figure S33. ^{13}C NMR spectra of 6-amino-9-benzyl-2-(2-methoxyethoxy)-7*H*-purin-8(9*H*)-one (**9**)

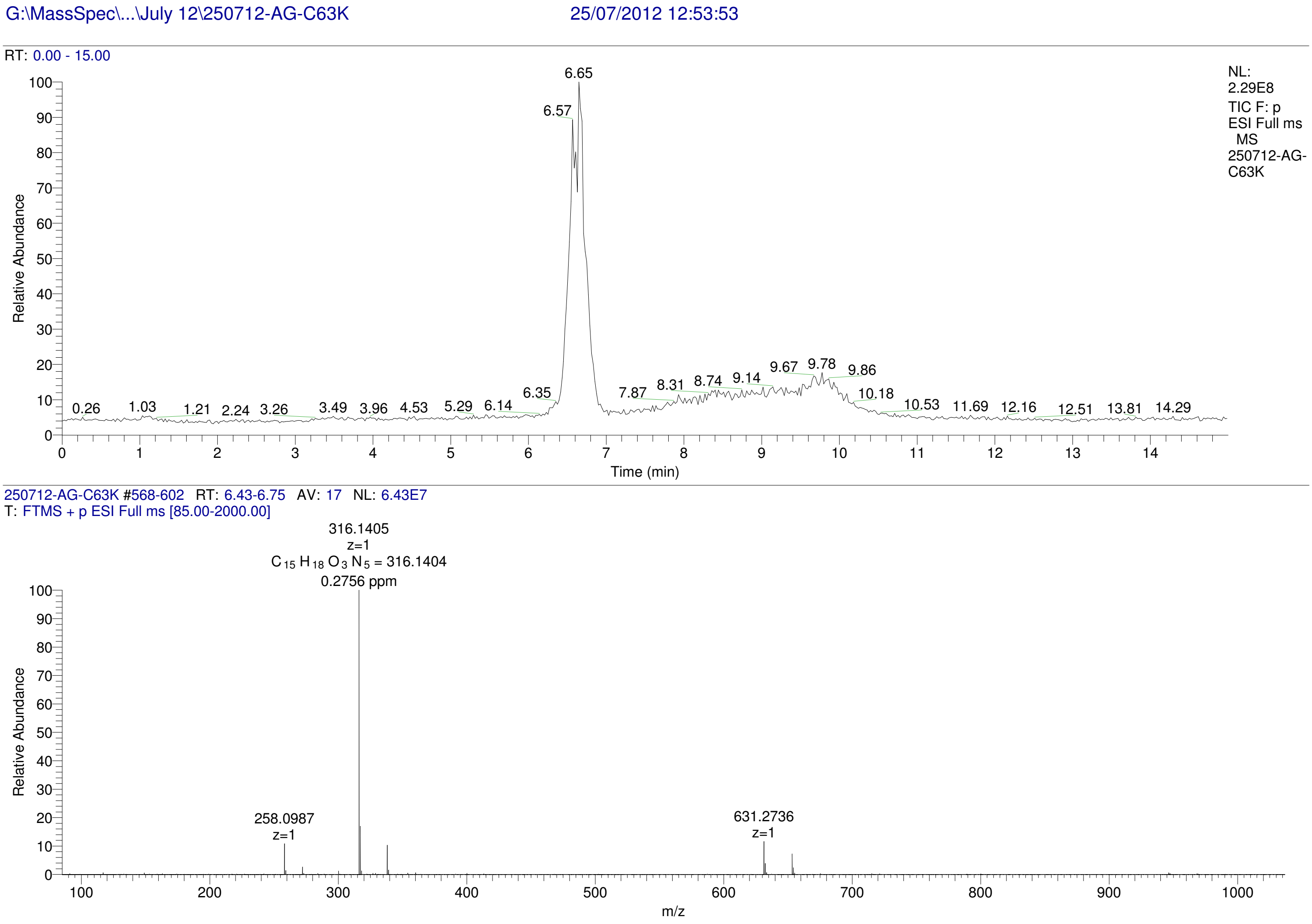

Figure S36. HRMS spectra of 6-amino-9-benzyl-2-(2-methoxyethoxy)-7*H*-purin-8(9*H*)-one (**9**)

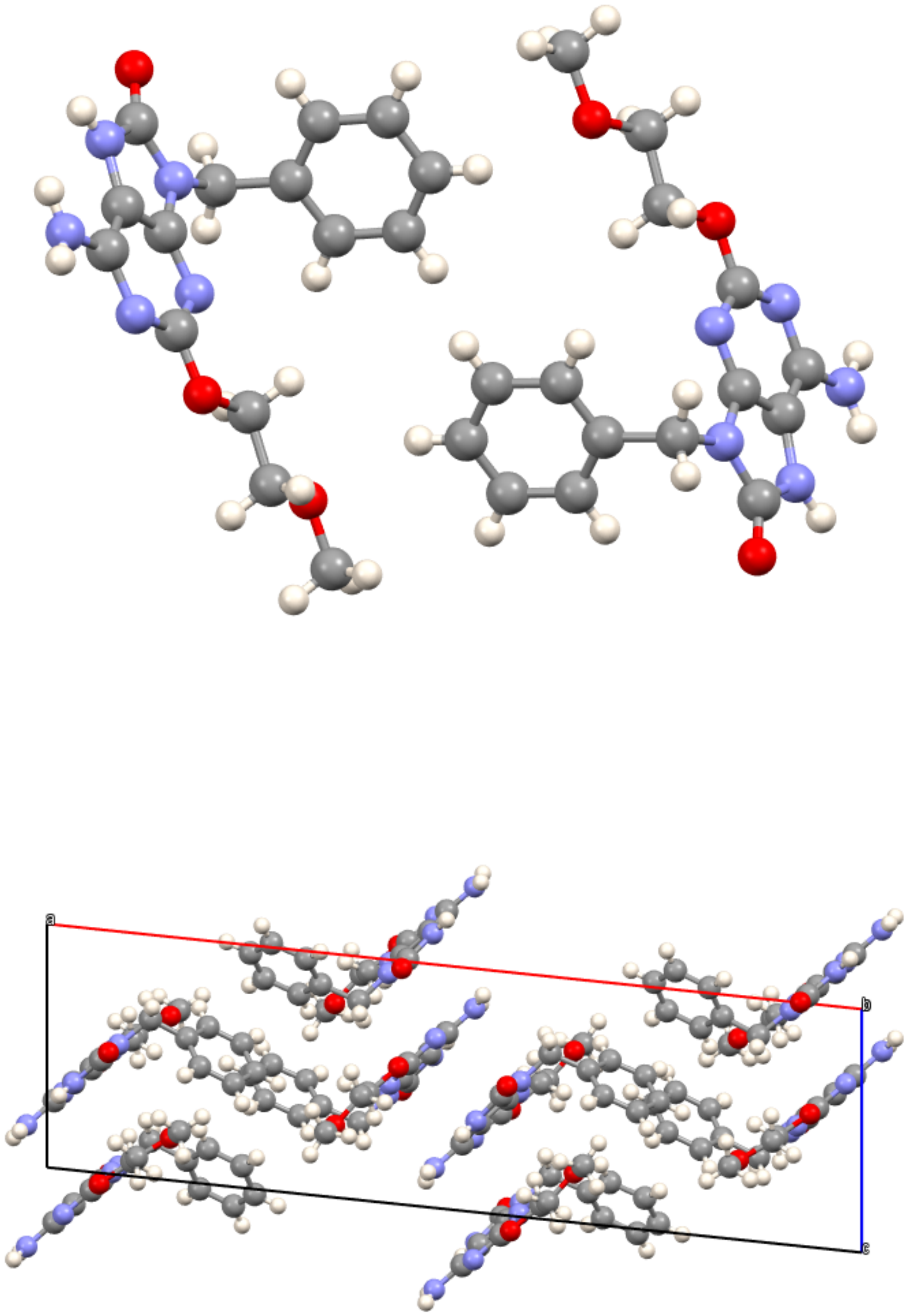

Figure S37. The two molecules of the asymmetric unit of **9** (upper) and the crystal packing of **9**, as viewed down the b-axis. Full details of the crystal structure are contained in the CIF that has been deposited (deposition number 1825910) with the Cambridge Crystallographic Data Centre.
